# Gender operationalisation and stress measurement in research with adolescent males: a scoping review

**DOI:** 10.1186/s12889-022-14351-x

**Published:** 2022-11-15

**Authors:** Parise Carmichael-Murphy, Ola Demkowicz, Neil Humphrey

**Affiliations:** University of Manchester, Manchester, UK

**Keywords:** Gender, Stress, Measurement, Operationalisation

## Abstract

**Aim:**

Stress measurement in adolescent males is in its relative infancy, which is likely to influence the effectiveness of mental health services for this heterogeneous population. Although evidence suggests the prevalence of mental health difficulties increases during adolescence, the relationship between gender and stress measurement is less explored or understood. This review summarizes findings on gender operationalisation and stress measurement in research with adolescent males.

**Methods:**

For this scoping review, six electronic databases across social and life sciences were searched using terms linked to adolescence, male, stress and research design. Articles were screened, data were extracted, and a narrative synthesis used to characterise studies by research design, adaptation of method for participants’ cultural context, operationalisation of gender, and measurement of stress.

**Results:**

Searches identified 3259 citations, 95 met inclusion criteria and were reviewed. Findings suggest that research on psychological stress in adolescence is a developing field, but one that is currently dominated by Western studies. Furthermore, the results indicate that stress measurement research with adolescent males tends not to make adaptations relative to participants' gender, age, or context.

**Conclusions:**

Stress research with adolescent males is lacking in scope. This review highlights the need for researchers to consider stress responses as more than a biological response, as it has been conceptualised historically. Recommendations for researchers to report research design and protocol more clearly are made to support readers to understand how stress and gender have been operationalised and measured and how this may influence research methodology. Future research should avoid conflating biological differences with gendered experience and demonstrate greater sensitivity to how gender identity may intersect with age and location to perpetuate gendered inequalities.

**Supplementary Information:**

The online version contains supplementary material available at 10.1186/s12889-022-14351-x.

## Background

One in seven people between ages 10–19 experiences a mental condition [1], with one-half of all mental illnesses developing by age 14 and two-thirds of mental illnesses develop by age 24 [2, 3]. For males, early-onset mental illness is linked to poor mental health across the lifespan [4]. Males in the UK are less likely to receive treatment or diagnosis for mental health [5], yet are three times more likely than females to end their life by suicide [6]. The risk of dying by suicide increases during adolescence, with the rates of completed suicide higher among young males than young females [7]. Exposure to stressful situations is linked to increased suicidal ideation [8], yet researchers know little about the role of gender-sensitive interventions for stress and suicide prevention with adolescent males specifically [9].

In Western cultures, those who are assigned male at birth are typically socialised and encouraged to perform gender in line with masculine norms which embody hegemonic ideals of inexpressiveness, emotional control, and low engagement with help-seeking [10]. Adolescent males in the Western context are typically socialised to adhere to hegemonic masculine ideals that promote strength and rationality, whilst avoiding vulnerability and expression of emotion [11]. For adolescent males, expectations to conform to masculine ideals encourage them to display physical and emotional strength, as well as to demonstrate autonomy and competency in dealing with mental distress [12]. Individuals who conform to high traditional masculine gender norms are at an increased risk of experiencing suicidal ideation and less likely to demonstrate help-seeking behaviour [11].

In the Western context, stress has historically been investigated from a physiological standpoint to determine how, when, and why disease occurs. This includes: risk factors associated with disease; protective factors to aid prevention; and, in particular, the role of stress in underlying etiological mechanisms [13]. Research indicates that factors such as genetics, culture, economics, and history influence individual reactivity to stress and shape how communities perceive stressors and ‘appropriate’ responses to them [14]. In other words, a stressor in one culture does not necessarily induce the same response in another culture, or even constitute a stressor at all. Considering the lack of clear definition, for the purpose of this systematic literature review, we focus on stress loosely as a psychological response to a stressor, rather than a biological or physiological operationalisation of stress that restricts understanding to a bodily response.

Stressors are considered to *cause* stress. Exposure to strong and persistent stressors that evoke stress responses are associated with the development of both physical and psychological illness [15]. Exposure to more stressors during adolescence is considered to negatively affect physical and mental health, and chronic stress is associated with greater propensity to develop mental health disorders including depression and anxiety [15]. Despite this, stress research is typically measured across the lifespan in four stages: in utero, childhood, adulthood, and lifespan, and often demonstrates a focus on differentiating stress during childhood *from* stress during adulthood [14]. The literature on stress mostly represents populations with chronic illnesses or diagnosed disorders in health settings [16], suggesting that male experience of distress without formal diagnosis across a range of settings and social contexts may be lacking. This has somewhat contributed to the lack of scope in recognition of young people’s experiences during the transition from childhood to adulthood specifically and possibly devaluing adolescence as a critical period in the lifespan for mental health intervention.

The term ‘stress’ is employed across both physical and mental health discourse to describe individual health and disease [17]; its use typically varies by the discipline in which it is used [18]. Although stress occurs at and across multiple levels of experience, efforts to measure this often fail to consider it beyond a biological response, focusing on physical symptoms or responses. Stress has proven a difficult concept to measure since it is considered ‘an emergent process’, involving ‘interactions between individual and environmental factors, historical and current events, allostatic states, and psychological and physiological reactivity’ [14]. Stress influences personal wellbeing across the lifespan; exposure to strong and persistent stressors is associated with the development of physical and psychological illness [14]. Although understood to impact health across the life course, there is a sense of uncertainty around what aspects of, or how much exposure to stress is detrimental to health.

Both sex and gender are typically conflated in epidemiological research. ‘Male’ is a term used to distinguish between biological sex differences that are assigned to an individual at birth. ‘Man’ is a term used to distinguish between gender identity differences; this is often self-identified [19]. Masculinity is used in reference to ‘patterns of expected behaviours that cultures use to construct generally accepted meanings of ‘being a man’’ [20]. Much of the literature examines and explores gender identity and masculinity in Western contexts; this is not surprising given the overrepresentation of Western populations in research [21]. Despite some greater recognition of gender identity beyond the binary in current policy and practice, in many instances, there is resistance to the appreciation of gender identity as a spectrum in defence of gender as a binary construct [10].

Quantitative researchers tends to present sex (male or female) and gender (man or woman/ boy or girl) as binary measures interchangeably [19]. In response, Lindqvist et al. (2020) deconstruct ‘gender’ into four ‘gender facets’: (i) physiological/bodily aspects; (ii) gender identity; (iii) legal gender; and (iv) gender expression. Appreciating gender as multi-faceted is vital to recognising *how* gender influences, or is related to, outcomes [19]. Conflating sex with gender (male = man) is likely to disguise *how* gender influences mental health outcomes and reduces the reliability of research.

In epidemiological studies, gender is typically employed as a binary variable without any researcher description of what exactly is mean by ‘gender’ for the means of the study. In stress research particularly, gender has been relied on as a quantifiable variable, used to predict a given outcome [19]. Measurement bias in standardised tools contribute to differences observed in mental health prevalence between males and females, including an increased likelihood of males receiving an incorrect or non-diagnosis because their symptoms are less recognised [9]. In this case, observed sex or gender differences in epidemiological studies should not automatically be valued as indicative of a need for sex-differentiated diagnostic materials, but rather as insight into the ways that gender-related coping and expression can be shaped by culture [5].

### The current study

To date, no study has synthesised how stress and gender are utilised in studies of adolescent males, but the results of such a study have the potential to inform research, policy, and practice to reduce stress-related mental ill health during adolescence. This scoping review examined studies that measure stress in adolescent males and how they operationalise gender in research. ‘Male’ is used throughout this review in acknowledgement that it is a term that has been normatively operationalised in scientific research as a marker of biological sex; however, ‘male’ is not used by the authors in assumption that it is synonymous with a person’s gender identity. Instead, male is adopted in this review to consider specifically how this population’s experiences of stress have been conceptualised across the research historically. This study intends to inform mental health policy and practice that impacts adolescents, contributing further to research on disparities in mental health as they are distinguished between ‘males’ and ‘females’. The review sought to:

(i) characterise trends in the study of stress measurement in adolescent males globally;
(ii) identify how stressors for adolescent males are measured across the literature;
(iii) discover how researchers operationalise gender in adolescent male stress research; and,
(iv) consider how adaptations to research design are made for participants’ age and cultural context.

## Method

Scoping literature reviews are useful for appraising the literature to identity gaps in knowledge and assessing the execution of research in a specified area [11, 22]. Arksey and O’Malley’s [23] methodological framework for scoping studies details six stages: (1) identify the research question; (2) identify relevant studies; (3) study selection; (4) chart the data; (5) Collate, summarise, and report the results, and a final optional stage, (6) consultation. This scoping review characterises international literature on gender operationalisation and stress measurement in research with adolescent males. An a priori protocol was developed to outline inclusion and exclusion criteria, as well as a search strategy. Six electronic databases across social and life sciences were searched systematically using terms linked to adolescence, male, stress and research design. Articles were screened, data were extracted, and a narrative synthesis used to characterise studies by research design, adaptation of method for participants’ cultural context, operationalisation of gender, and measurement of stress.

### Eligibility criteria

The outcome of interest was the measurement of psychological stress in epidemiological studies, with male participants aged 10–24 years, including studies where the total sample or a disaggregated sample had a mean, median, or upper range age of no less than 10 and no more than 24.99. Studies that observe males and females together where the data for males is disaggregated were included, but a comparison with females was not intended (given our stated intention to focus specifically on males). Studies that include any person who self-identifies as male, boy, or man were included in this review and the operationalisation of gender in studies is reported.

Only studies originally written in English were included as the translation of those published in alternative languages may compromise the intended comprehension of the text. Studies published before 1991 were excluded due to the inconsistency in the categorisation of clinical trials before the introduction of indexing and classification requirements, which means that the likelihood of identifying all relevant studies before this date is not reliable using electronic databases [24]. This review considered all primary epidemiological study designs. Study design was included in the search strategy to narrow down findings to studies relevant to the research questions, study designs included are adapted from the methodological framework in epidemiology [25]. Only manuscripts published in peer-reviewed outputs were consulted for this review as grey literature, such as dissertations and working papers do not routinely undergo the peer-review process [26]; for this reason, their study classification may be less consistent and comparable.

### Search strategy

Databases PsychINFO, PubMED, Scopus, CINAHL, ProQuest, and Web of Science were systematically searched on 9^th^ March 2021. Search parameters were restricted to the English language only and from the years 1991 to that date. Search strings were adapted to each database using Boolean operators and truncation where possible. An example search string is presented in Table 1 and a full search strategy is available in Supplementary Document A.

**Table 1:**
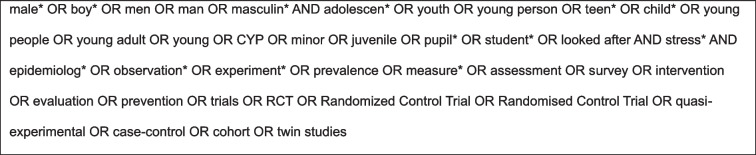
Example search strategy

### Study selection

Articles were selected following the Preferred Reporting of Items for Systematic Reviews and Meta-Analyses (PRISMA) Statement [16,27]. All search results were downloaded into Mendeley reference management software where duplicate citations were removed. Of the total hits, 1% were randomly selected and screened by PC-M, OD, and NH for calibration purposes with full consensus reached. PC-M independently screened the rest of all hits by title and abstract against the eligibility criteria. NH spot-checked 5% of the articles considered eligible for the review and was in full consensus with PC-M.

**Fig. 1:**
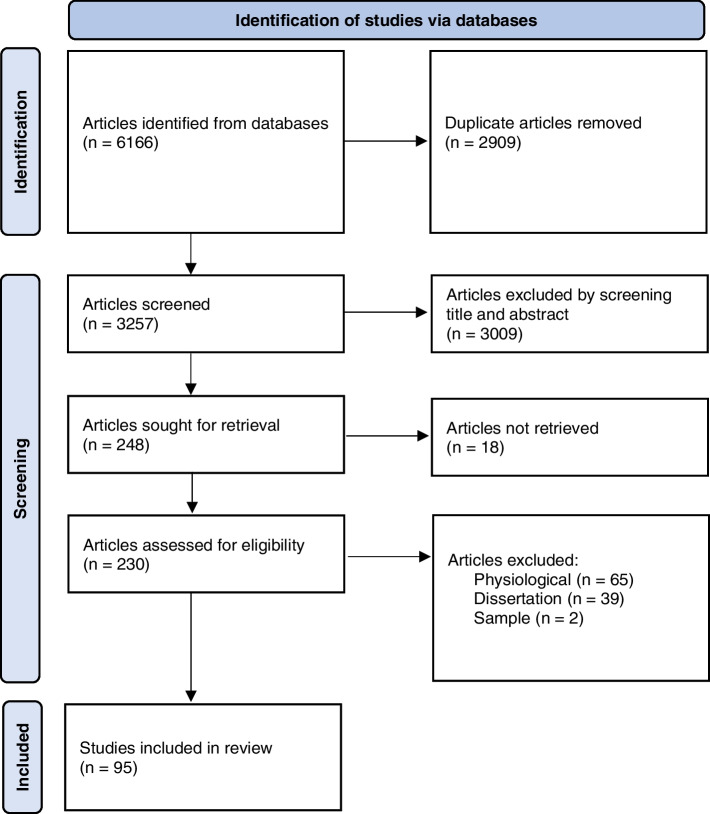
PRISMA flowchart of review

The literature search produced 3257 articles across six databases. After removing duplicates and screening title and abstract, 248 full texts were reviewed. 124 full texts were excluded because they were either: unavailable; primarily physiological or biological studies of stress; non-peer reviewed dissertation; and/or inappropriate populations. 29 articles were removed after screening for explicit measurement of stress in the method section, leaving 95 articles eligible for the synthesis. Search results and the screening processes are presented in the PRISMA flow chart in Fig. 1.

### Data charting

Full-text articles were obtained and assessed against the eligibility criteria by PC-M. Where access to texts was restricted by paywalls, PC-M contacted first authors of articles via ResearchGate or by email. If no reply was received from the author within six weeks, the study was excluded from this review as ‘unavailable’. PC-M extracted information from each full text and recorded for synthesis of the studies using a template created in Microsoft Word. This included: (i) study details: ID, year, country, research design, age of sample, total sample and percentage of adolescent male sample; (ii) conceptualisation of stress: scale or tool used, outcome measured; and (iii) operationalisation of gender: gendered participant term, gender facet.

Twenty one studies with unstated and unclear research design were checked by NH which highlighted the need to distinguish experimental studies further as either (1) clinical trial: randomised trial of a prescribed intervention; or (2) laboratory: experimental procedure involving manipulation of independent variable(s) to ascertain impact on dependent variable(s). Study design was not always explicitly stated in the full text articles so OD spot-checked 20% of the study research designs for accuracy and was in full agreeance with PC-M’s categorisation.

Studies were assessed for whether they had made adaptations to the study design for participants’ age or cultural context. The methods section of each article was considered to determine whether researchers stated explicit research adaptations, whether adaptations were self-evident, the protocol was designed to accommodate participants, or whether no adaptation were reported.

### Data synthesis

It was anticipated that stress data would be collected using a range of observational and experimental methods, measurement tools and participant groups. Accordingly, a narrative synthesis approach was considered to be the optimal means of synthesising findings from a wide range of studies. Meta demographic statistics including mean age of sample and mean percentage of males from the total sample are presented. Where studies reported a mean age for participants, a total mean age was calculated. Where studies included male and female samples, the percentage of male participants was calculated, and a total mean percentage of male sample was calculated.

## Results

This scoping review sought to identify and synthesise published research on stress in adolescent males to explore trends in the measurement of stress across age and context. Results of the review are presented below:

### Characteristics of included studies

**Fig. 2:**
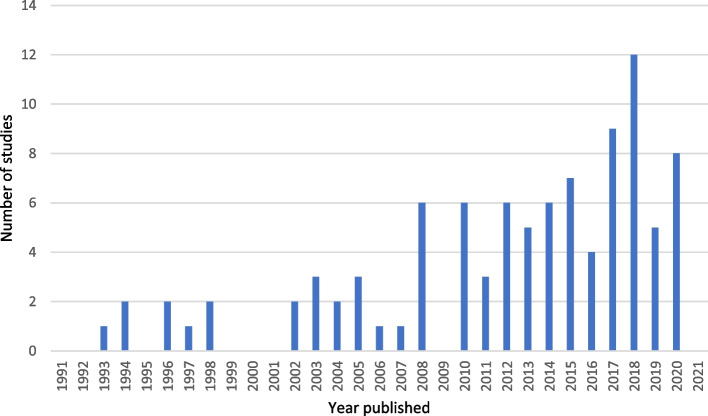
Number of studies published by year

A total of 95 epidemiological studies of stress in adolescent males were identified for this review; characteristics of each are provided in Supplementary Document A**.** Studies were published across the decades are as follows: 1990–1999 (8.3%); 2000–2009 (18.2%); 2010–2019 (65%); and 2020 + (8.3%). Figure 2 shows the number of studies published by year.

**Fig. 3:**
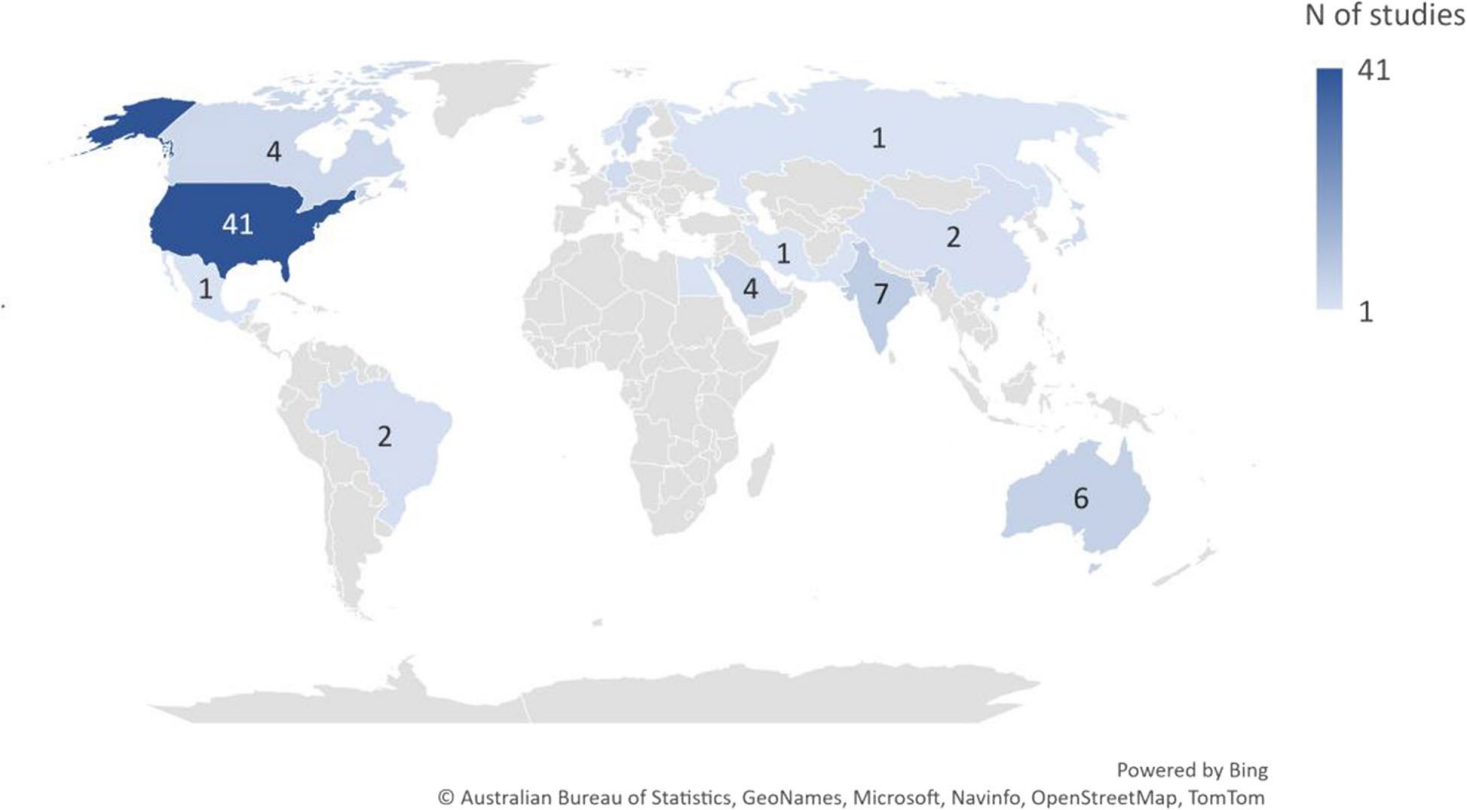
Number of studies across countries

Reviewed studies included samples from 24 countries, across six continents. Over two-fifths of the studies (*N* = 41; 43.1%) were conducted in the United States. Just over one-fifth of the studies were conducted outside of the Americas, Europe, or Australasia. Figure 3 shows the spread of studies across countries.

The average sample size was *N* = 562 with a range of *N* = 15 to 5,308. 42 studies reported a mean age for participants; the average age of participants across these studies was 20.89, with a range of 11.09 to 24.98. 35 studies included both male and female samples; of these, the average proportion of male participants was 48.1% with a range of 8% to 99.1%. Samples were drawn from a range of settings including; educational contexts, community spaces, online platforms, detention centres, and the general population. Some studies drew their sample from a combination of settings. More than half of the studies (*N* = 53; 55.8%) recruited all or part of their sample from educational settings such as schools, colleges, and universities; with 22 (23.1%) studies drawing all or part of their sample from a university.

### Report of key findings

Key findings from the 95 studies are explored concerning their: assessment of research design; operationalisation of gender; and measurement of stress. In brief, the research design of the study was not always explicitly stated. Further still, the majority of studies did not disclose or make adaptations for participants relative to their age or cultural context. Only four studies used less gender normative terminology, with more explicit reference to participants’ biological facet of gender. Only nine of the identified studies sought to measure a stressor.

#### Measurement of stress

Studies drew upon a variety of measurement methods and tools to examine stress as a process, but only nine (9.5%) studies described an intention to measure stressors as a cause in the research protocol. Examples include using the: ‘Life Events Checklist’ to measure recent episodic stressors [28]; ‘Life Stress Interview’ to measure chronic stressors in the past year [29]; ‘Adolescent Stress Questionnaire’ to measure exposure and appraisal of stress over five years [30]; ‘Urban Hassles Index’ to measure stressors in the urban environment [31]; and the ‘Racist Hassles Questionnaire’ to measure young adult contextual stressors [32]. 8 (8.4%) studies mentioned ‘stressor’ in the title with no reference to the measurement of a stressor in the research protocol.

The most popular measurement tool used across articles was the Perceived Stress Scale (PSS), used in 16 (16.8%) studies. Of the 16 studies using the PSS: nine studies used the 10 item scale; three used the 14 item scale; one used the 22 item scale; and three studies did not specify how many items the scale had. See Table 2 for a list of these 16 studies.

**Table 2:** Studies and versions of the PSS scale used

PSS scale used	Studies
10 item	[33–41]
14 item	[42–44]
22 item	[45]
Not specified	[46–48]

Eight (7.6%) studies used the Masculine Gender Role Stress (MGRS) scale to examine men’s experiences of stress relating to masculine identity or male gender role expectations. 4 (3.8%) of studies used the Depression Anxiety Stress Scale-21 (DASS-21) to measure symptoms of depression, anxiety, and stress. A full list of tools used to measure stress across the 95 studies is provided in Supplementary Document A.

#### Operationalisation of gender

Of the 95 articles, 48 (50.5%) recorded participants as ‘males’ which is a physiological descriptor, whilst eight (19%) recorded participants as ‘boys’ and 14 (14.7%) described participants as ‘men’ which are social descriptors. A further 10 (10.5%) studies recorded participants using ‘boys’ and ‘males’ or ‘men’ and ‘males’ interchangeably. A small subset of studies (4.2%) published between 2017–2019 utilised less gender normative terminology to describe participants' gender(s) with two studies using ‘assigned male at birth’, one study using ‘biologically male and male-identified’, and one more study used ‘born male’ and ‘men’ interchangeably. See Table 3 below for a list of these four studies.

**Table 3:** Characteristics of studies using less gender-conforming terms

Country	Design	Sample age	Gendered term used	Title descriptors	Reference
US	cross-sectional (observational)	23 (median)	born male & men	young Black men who have sex with men	[49]
US	cohort (observational)	22.85(mean)	assigned male at birth	Young male same-sex couples	[40]
US	cohort (observational)	23.06 (mean)	assigned male at birth	Young male same-sex couples	[41]
Lebanon	cohort (observational)	23.9 (mean)	biologically male & male-identified	Young Men Who Have Sex with Men	[50]

All four of these studies [40, 41, 49, 50] sought to measure stress in participants who were categorised by their sexuality, typically described in these studies as ‘men who have sex with men’ or ‘male same-sex couples’. These four studies consisted of 100% male samples with average ages ranging from 22–24 and an average sample size of *N* = 315 but ranging between 109 to 618 participants. These four studies were published between 2017 and 2019, suggesting that research that embraces less gender-conforming terminology is in its relevant infancy.

#### Assessment of research design

More than three quarters (*N* = 80, 76%) of studies were observational, with more than half (*N* = 57, 54.1%) of the total studies adopting a cross-sectional design. 15 studies adopted an experimental design; only 3 of which were clinical trials, and all conducted in the United States. Table 4 shows the total 95 studies by research design.

**Table 4:** Frequency and percentage of studies by research design

Study type	Number of studies (% of total)	Research design	Number of studies (% of total)
Observational	80 (76)	Cross-sectional	57 (54.1)
		Cohort	20 (19)
		Case–control	3 (2.85)
Experimental	15 (14.25)	Clinical trial	3 (2.85)
		Laboratory	12 (11.4)
**Total**	**95 (100)**		**95 (100)**

Across the research corpus, study design was not often stated explicitly in the title or abstract; this was particularly more notable for experimental studies. In many cases, the design of the study was not explicitly stated until drawn upon as a limitation; for example, that a cross-sectional design would be complemented by more longitudinal research.

Across the articles, less than one third (*N* = 28, 29.5%) of studies disclosed adaptation for the age of participants in the method section. Only one (1%) study [51] disclosed that the measure used (DASS-42) was adapted or intended for participants’ age group *and* cultural context. In some cases, it was evident from the name or description of the measurement tool that it was intended for use with adolescents. Examples of self-evident measures relative to participants’ age include: Escala de Stress Infantil (ESI) (Child Stress Scale) [52], Adolescent Life Event Stress Scale [53], Children’s PTSD Inventory [54], and Trauma Symptom Checklist for Children-PTS section [55]. Examples of self-evident measures relative to participants’ cultural context include: Hindi adaptation of Student Academic Stress Scale [56], Arabic version of DASS-42 [51], German Version of the Positive and Negative Affect Schedule [57], and Chinese version of the Depression Anxiety Stress Scale-21 (DASS-21) [58].

Table 5 and Table 6 show studies categorised as: (i) no evidence of adapted research design for participants' age or cultural context; (ii) evidence that the process or protocol was designed to accommodate participants’ age or cultural context; (iii) the measure used is acknowledged as adapted or intended for the age group or cultural context of participants; (iv) or that is was self-evident in the measure’s name or description provided that it is considered appropriate for the age or cultural context of participants.

**Table 5:** Categorisation of studies that made adaptations for age of participants

Level of adaptation	Examples of adaptation taken from studies	Number of studies (% of total)
No evidence in method that design was adapted for participants’ age	n/a	67 (70.5)
Process or protocol designed to accommodate participants’ age	Pilot study to obtain feedback about the clarity, length, comprehensiveness, and time of completion of measures [42]; Items deleted, added, or changed to make more appropriate for children’s self-reports [59]; Items adapted to address the adolescent’s situation [60]; Modifications to instrument based on judgement of research team and focus groups [61]; Items read aloud and paraphrased and participants encouraged to ask questions when uncertain of item meaning [62]	4 (4.2)
Measure acknowledged as adapted or intended for age group (designed or validated)	Good internal consistency and test–retest reliability among adolescents [28]; PSS has demonstrated good internal consistency in both adolescents [35]; Urban Hassles Scale has adequate psycho-metric properties for adolescent respondents [28]	6 (6.3)
Self-evident in name or description that measure is appropriate for age group	Adolescent Minor Stress Inventory [63]; Child Stress Scale [52]; Trauma Symptom Checklist for Children-PTS section [55]; 40-item Adolescent Life Event Stress Scale [53]	18 (19)

**Table 6:** Categorisation of studies that made adaptations for cultural context of participants

Level of adaptation	Examples of adaptation taken from studies	Number of studies (% of total)
No evidence in method that design was adapted for participants’ cultural context	n/a	72 (72.7)
Process or protocol designed to accommodate participants’ cultural context	Translation, back-translation, and retranslation into Spanish [64]; Survey administered in English or Arabic dependent on participant preference [50]; Providing guidance in local language (Urdu) to convey exact meaning of questions [45]	8 (8.4)
Measure acknowledged as adapted or intended for cultural context (designed or validated)	Validated Chinese version of the DASS-21 [58]; Validated in Arab population [33]; Hare Area Specific Self-Esteem Scale developed specifically for African American adolescents [65]; Australia DASS-21 validated in both clinical and nonclinical samples [66]	6 (6.1)
Self-evident in name or description that measure is intended for cultural context	Korean version of the Coping Style Questionnaire [67]; Arabic version of DASS-42 [51]; Japanese Perceived Stress Scale [46]; Brazilian Portuguese version of the PSS [38]	13 (13.7)

Four (4.2%) studies stated that the process or protocol was designed to accommodate participants’ age. Adaptations include: ‘concerted efforts to develop items that could be conceptualized’ by participants relative to their level of language and reading [64]; ‘items are adapted to address the adolescent’s situation’ [60]; and pilot studies to ‘obtain feedback about the clarity, length, comprehensiveness, time of completion’ [42].

Of the total, 9 (9.1%) studies stated that the process or protocol was designed to accommodate participants’ cultural context. Examples of how processes or protocols were adapted include: ‘translated, back-translated, and retranslated into the Spanish forms most likely to be understood by the Hispanic students’ [64]; providing ‘guidance in the local language (Urdu) to convey the exact meaning of the presented questions’ [45]; scale translation ‘from English into Albanian and checked through back-translation to English to establish equivalence in translation’ [68]; and survey administration in ‘English or Arabic, depending on the preference of the participant’ [50].

## Discussion

The aim of this review was to explore how stress is measured and how gender is operationalised in stress research with adolescent males. We also sought to investigate how stressors for adolescent males are measured across the literature, as well how adaptations to research design are made for participants’ age and cultural context. The results of this scoping review contribute to the growing body of literature on adolescent males’ mental health and wellbeing. 95 studies were eligible for review that sought to measure stress in participants described as ‘male’, ‘boy’, or ‘man’ between ages 10–24. The review highlights that stress research with adolescent male populations is in its relative infancy, with the majority of studies identified published during the last decade and within the United States.

The review highlighted that in studies in this area, most researchers did not acknowledge or make accommodations to their protocol for participants’ age. Given the uncertainty around the specific demarcation point between childhood and adulthood, the introduction of ‘young’ and ‘emerging’ adulthood as terms used in reference to the transitionary period between adolescence and adulthood [69] is somewhat reflected in the reviewed literature. Although ‘young’ was used frequently to describe participants (26 studies), only six of those studies made specific reference to participants as ‘young adults’; only two of the total reviewed studies used ‘emerging’ to describe their participants. Sawyer et al. [69] push for ages 10–24 to be considered under the umbrella of ‘adolescence’, particularly in response to earlier onset puberty, legal requirements to extend to school leaving age, access to training and employment, as well as social expectations in line with media and technological developments.

Often ‘psychological distress’ is used broadly to describe symptoms of stress, anxiety and depression, with higher levels of psychological distress considered indicative of mental ill-health [14]. In the UK, the ongoing COVID-19 pandemic is likely to impact negatively on children and young people who have experienced school closures, exam cancellations, illness and bereavement [70, 71]. In addition to a decade of austerity across the UK that has vastly restricted access to youth services [72] the National Health Service, plus the introduction of compulsory education from age 16 to 18. It could therefore be argued that the boundaries between childhood and adulthood are blurring, and adolescence as a timepoint is open to interpretation.

The findings illustrate a recent shift toward more gender-inclusive approaches. Scientific researchers are beginning to acknowledge and make reasonable adjustments to their research protocol to ensure that the research approach is inclusive of gender diverse and gender non-conforming populations. However, of the four eligible studies that were identified as using less gender normative language (e.g. ‘assigned male at birth’), the mean age of participants ranged between 22.85–23.9. This is at the top end of the eligibility for this review and highlights that gender-sensitive stress research in school-age children and young people is lacking. Further still, these four studies used terminology to describe participants’ sexuality in the research title, such as ‘men who have sex with men’ and ‘male same-sex couples’. Researchers must remain critical of terminology used in research and its historical context; in this case, how the removal of homosexuality from the Diagnostic and Statistical Manual of Mental Disorders (DSM) initiated a shift away from identifying a cause and subsequent treatment of homosexuality [73]. This is interesting since the review highlights that few studies sought to explore common stressors for adolescent males (what causes the stress), instead focusing on their perception or response to the stress. When researchers fail to acknowledge gender identity as a spectrum, that is not synonymous with biological sex, they are less likely to recognise their likelihood to misinterpret findings that rely on gender as a dichotomous variable. And further still, researchers who offer pre-defined responses are discriminating against participants who are denied the opportunity to self-define their own gender identity [19]. In this sense, it could be argued that researchers are supporting and contributing to the construction of gender as a binary variable, whether they agree with that or not.

The review found that only nine studies examined stressors in adolescence; all of which were published after 2010, with six of these from the United States. These nine studies tended to measure stressors relative to time (i.e., ‘episodic’, ‘chronic’, and ‘life’), as well as place (i.e., ‘urban environment’, ‘contextual’, and ‘social-environmental’). It could be argued that this indicates a lack of intention to consider the contextual and subjective nature of experiences of stressors, and an attempt to objectively compare them relative to time and space. As such, the possibility of working toward a more ‘cumulative science’ that adopts a longitudinal approach to understanding the impact stressors across the lifespan is restricted. This would be particularly useful in the shift toward greater recognition of how socioeconomic factors such as childhood poverty and family income accelerate health inequities amongst childhood and adolescence neural functioning, stress dysregulation, and mental health [74–76].

The literature review showed the Perceived Stress Scale (PSS) as the most popular measure; this is not surprising given that it is relatively brief and intended for use as a broad index of perceived stress. Across the studies identified in this review, the PSS was typically used as a measure of subjective stress, but in some cases, researchers noted that the tool was used to examine academic stress, sources of stress, lack of control or worry about meeting demands specifically. Despite the popularity of the PSS as a measure of stress, it has two limitations that are particularly important to consider. Firstly, the PSS does not measure cumulative experience, focusing more on recent experiences; so, they are less reliable as a predictor of future health [14]. Over half of eligible studies were cross-sectional in design, which is highly susceptible to researcher bias [14]. It could then be argued that a reliance on the PSS in research with adolescent males is restricting the ability to inform long term provision. Secondly, subjective reporting can be inhibited by individual ability and circumstance to disclose personal experiences of stress [14].

The second most popular tool used with this population was the Masculine Gender Role Stress Scale (MGRS); designed to measure men’s appraisals of stressful situations [77]. For adolescent males specifically, disclosing personal experiences of stress could be impeded by societal expectations for males to conform to high traditional masculine gender norms and avoid help-seeking [30]. Males in Western culture across the lifespan are encouraged to ‘perform’ gender in line with hegemonic masculine norms that embody ideals of inexpressiveness, emotional control, and low engagement with help-seeking [77]. It is not so clear why people ascribe to traditional masculine ideologies. Still, it is important to acknowledge that it is not necessarily ‘masculinity’ that is the issue, but the adoption of masculinity that adhere to hegemonic belief systems and practices. Instead, researchers are encouraging interventions that support more ‘healthy;’ expressions of masculinity and masculine identities than the traditional hegemonic masculinities that dominate socialisation in Western countries [77].

Our review identified that the majority of studies did not state explicit adaptations or considerations of suitability in the method section for the age (70.5%) or geographic location (72.7%) of participants. Although some studies did make adaptations in the protocol for participants’ age (*N* = 28) or cultural context (*N* = 23), the overall 95 studies suggest that this is not common. Although written from an English-speaking context, the findings here offer an opportunity to consider global trends in stress measurement for adolescent males. This is particularly pertinent given that the COVID-19 pandemic will likely continue to impact young people’s education, employment and health prospects for the foreseeable future.

There is a rapidly growing body of literature that considers adolescent mental health in light of the global COVID-19 pandemic which may indicate a shift toward recognising the interconnectedness of adolescents’ wellbeing as linked to access to education, economic, and health resources. Researchers should also consider the historical context in which mental health is conceptualised and applied to describe or categorise individual and group experiences. An historical emphasis on ‘stress’ as a biological response and the use of ‘male’ or ‘female’ as biological categories has contributed to a biological overdetermination of mental health. Robinson [18] highlights that the diversity of stress research applications across disciplines contributes to inconsistency in the application of the term ‘stress’ in research and that largely, this is due to a lack of historical awareness of how the concept of stress developed. Findings of this review suggest that research that seeks to explore the relationships between adolescent males’ gender and experience of stress are outdated, and does not capture adolescents who are assigned male at birth experiences of stress today.

### Recommendations

This scoping review highlighted that the body of stress research concerning adolescent males tends not to report considerations on the relevance of research tools and protocol for participants relative to their age and cultural context. As such, we argue that researchers should be more specific when reporting both stress [78] and gender [79]. More care is needed with which aspect of stress and gender researchers are exploring, so they can be clearer in their own reporting and so readers can more confidently interpret findings.

It is not enough to state that gender predicts an outcome, but *where*, *when* and *why* gender comes to predict outcomes in a given scenario [19]. Researchers should recognise that biological sex as a proxy for gender identity contributes to recreating gendered inequalities and cultural biases. In this sense, researchers are by default supporting and contributing to the construction of gender, whether they agree with it or not [19]. There is a need to acknowledge when gender serves as a quantifiable category, but with consideration as to how this informs research questions and design; at the very least to acknowledge the limitations of this approach. To avoid measurement error, researchers might consider using a free-text response for participants to self-report their gender (see Lindqvist et al., 2020 for an empirical example [19]). Still, categorisation of free-text responses can be time-consuming although this should not automatically be given as a reason to avoid this approach. There will not be a ‘one-size-fits-all’ approach to operationalising gender in research; Fraser’s [79] flowchart for selecting a gender identity measure may serve as a useful tool for researchers to think through their selection of gender identity measures. Ultimately, researchers should demonstrate an understanding of *why* gender is important for their research question and *which* aspects of it they are attempting to record, measure, or control [19].

Researchers should also offer clarity on whether they are examining stress exposure, stress response, or both. Epel et al. [14] offer a Stress Typology that illustrates the conceptual dimensionality of stress. This typology includes details on i) stressor exposure characteristics, and ii) psychological and behavioural responses to specific stimuli or events. Researchers should consider how stress the measure of stress exposure and response can influence research conceptually and methodologically; in turn, this should help offer clarity to research questions. Stress measurement should be in line with the context in which it is employed, otherwise, its’ predictive ability is limited; Crosswell and Lockwood [78] detail ‘best practices’ for stress measurement, including a summary of steps for choosing appropriate stress measures. Researchers should consider the uniqueness of participants alongside how communities perceive stressors and stress responses, including how geographical and historical events influence local and global perceptions.

In this sense, researchers might be encouraged to think about what *else* might influence the outcome in addition to, or even instead of, gender. Researchers should account for how vulnerability to stressors changes across the lifespan, by choosing a measure that is appropriate for participants' developmental stage. Sawyer et al. [69] promote the more inclusive 10–24, rather than 10–19, age range for adolescence that enables researchers to consider experiences *within* the transition to adulthood, and not just as a specific unitary timepoint in the lifespan. As such, experiences of those categorised *within* gender groups are more likely to be considered, for example how age, race, disability, ethnicity, social class, income, language, religion, or sexuality can influence individual stress exposure and response.

### Strengths and limitations

This scoping review sought to provide an overview of research that measured stress in adolescent males. However, given that this review seeks to provide an overview of the literature as it represents adolescent males, authors acknowledge that ‘male’ is term is normatively employed to categorise diverse groups of people who have been assigned male at birth. Given the growing uncertainty around the age of adolescence, it is not possible from these studies to ascertain which age range should be used in reference to adolescent populations, but it does support that there is a general lack in the scope of studies for males across this timepoint. Lastly, this scoping review focused on stress solely; researchers may have adapted their design or protocol to explore different variables across age and location such as anxiety or depression but as this is not the focus of this review, any such adaptations are not reported.

## Conclusions

This scoping review sought to identify and synthesise published research on stress in adolescent males. The study found that most researchers did not acknowledge or make accommodations to their protocol for participants’ age or cultural context. Further still, this review contributes to the growing body of literature that encourages researchers to engage with ‘stress’ as more than a biological response. An overreliance on biological markers of difference in stress research must distinguish between populations overlooks how individual health might be influenced by experiences related to gender *identity*. It could be argued that the lack of sensitivity toward gender identity as it intersects with age and context leads mental health provision to perpetuate gendered inequalities by conflating biological differences with gendered experiences. Stress research with adolescent males is lacking in scope, particularly concerning methods used to explore experiences of stress in this diverse population. Clearer reporting of research design and protocol is needed to: (i) support readers to understand how stress and gender have been operationalised and measured, and (ii) support greater recognition of the role of culture in research design. It is hoped that more conceptual clarity in stress research will further awareness of ‘stress’ as an individual and personal experience that is not necessarily being captured well *enough* in research so far.

## Supplementary Information

**Additional file 1.**

## Data Availability

Example search strategy and full list of included studies are available in supplementary information.
