# Supplementary material for: Gender operationalisation and stress measurement in research with adolescent males: a scoping review
Source: BMC Public Health. 2022 Nov 15;22:2082. doi: 10.1186/s12889-022-14351-x (PMC9664422; doi:10.1186/s12889-022-14351-x)
Supplement: Supplementary file 1 — Additional file 1. [file 12889_2022_14351_MOESM1_ESM.docx]

**Supplementary Document A: Characteristics of included studies**

| **ID** | **Country (Year)** | **Research design** | **Sample drawn from** | **Age of sample** | **Total Sample**  **(% of adolescent males)** | **Scale or tool** | **Outcome measured** | **Gendered participant term**  **(gender facet)** | **Terms used in title to describe population** | **Reference** |
| --- | --- | --- | --- | --- | --- | --- | --- | --- | --- | --- |
| **1** | Pakistan (2016) | cross-sectional (observational) | schools, shopping centres & homes | 10-25 | 90  (92.2%) | Perceived Stress Scale | Perceived stress | males (physiological) | males of different ages | Abbas, N. (2016). The girlfriend’s effect on perceived stress in sample containing males of different ages from Attock and Sargodha, Pakistan: with moderating role of Islamic religious values. *Mental Health, Religion & Culture*, 19(7), 639–647. DOI: 10.1080/13674676.2016.1204275 |
| **2** | Saudi Arabia (2007) | cross-sectional (observational) | secondary schools | 15-19 | 1723 (100%) | Arabic version of Depression, Anxiety and  Stress Scale (DASS) | levels of depression, anxiety and stress | boys (social) | Saudi adolescent school boys | Al-Gelban, K. S. (2007). Depression, anxiety and stress among Saudi adolescent school boys. *Journal of the Royal Society for the Promotion of Health,* 127(1), 33–37. DOI: 10.1177/1466424007070492 |
| **3** | Saudi Arabia (2017) | cross-sectional (observational) | university | 18-23 | 213  (99.1%) | Perceived Stress Scale | academic stress | Males (physiological) | male pharmacy and medical students | Al-Shagawi, M. A., Ahmad, R., Naqvi, A. A., & Ahmad, N. (2017). Determinants of academic stress and stress-related self-medication practice among undergraduate male pharmacy and medical students of a tertiary educational institution in Saudi Arabia. *Tropical Journal of Pharmaceutical Research,* 16(12), 2997–3003. DOI: 10.4314/tjpr.v16i12.26 |
| **4** | Saudi Arabia (2013) | cross-sectional (observational) | university | M=21 | 214  (100%) | Dental Environment Stress (DES) questionnaire | Level of stress | males (physiological) | male undergraduate dental students | Alzahem, A. M., Van der Molen, H. T., & De Boer, B. J. (2013). Effect of year of study on stress levels in male undergraduate dental students. *Advances in Medical Education & Practice,* 4, 217–222. DOI: 10.2147/AMEP.S46214 |
| **5** | England (1993) | cross-sectional (observational) | university | M=22.82 | 204  (41.2%) | Masculine Gender Role Stress (MGRS) Scale | Stressful situations for men | males (physiological) | n/a | Arrindell, W. A., Kolk, A. M., Pickersgill, M. J., & Hageman, W. J. (1993). Biological sex, sex role orientation, masculine sex role stress, dissimulation and self-reported fears. *Advances in Behaviour Research & Therapy,* 15(2), 103–146. DOI: 10.1016/0146-6402(93)90018-W |
| **6** | India (2014) | cross-sectional (observational) | compulsory schools | 15-19 | 353  (100%) | Adolescent Life Event Stress Scale | stress due to life events experienced in the past year | boys (social) | Boys | Augustine, L. F., Nair, K. M., Rao, S. F., Rao, M. V., Ravinder, P.,  Balakrishna, N., Laxmaiah, A. & Vazir, S. (2014). Adolescent Life-Event Stress in Boys Is Associated with Elevated IL-6 and Hepcidin but Not Hypoferremia. *Journal of the American College of Nutrition*, 33:5, 354-362, DOI: 10.1080/07315724.2013.875417 |
|  |  |  |  |  |  | General Health Questionnaire (GHQ-12 | psychological morbidity or psychological distress |  |  |  |
| **7** | United States (2011) | cross-sectional (observational) | juvenile detention center | 12-17 | 83  (100%) | PTSD–RI | trauma exposure and PTSD symptoms | boys (social) | detained boys | Becker, S. P., & Kerig, P. K. (2011). Posttraumatic stress symptoms are associated with the frequency and severity of delinquency among detained boys. *Journal of Clinical Child & Adolescent Psychology,* 40(5), 765–771. DOI: 10.1080/15374416.2011.597091 |
| **8** | United States (2010) | cross-sectional (observational) | public schools | 10-20 | 254  (49%) | Urban Hassles Index | Stressors in urban environment | males (physiological) | young African American males | Bennett , M. D. J., & Olugbala, F. K. (2010). Don’t bother me, I can’t cope: Stress, coping, and problem behaviors among young African American males. Social Work with African American Males. *Health, Mental Health, & Social Policy,* 179–194. DOI: 10.1093/acprof:oso/9780195314366.001.0001 |
| **9** | United States (1994) | cohort (observational) | middle school | 11-13 | 2446 (100%) | Stressful life events questionnaire | Number of stressful life events encountered | boys (social) | adolescent boys | Biafora, F. A., Vega, W. A., Warheit, G. J., & Gil, A. G. (1994). Stressful life event and changes in substance use among a multiracial ethnic sample of adolescent boys. *Journal of Community Psychology*, 22(4), 296–311. DOI: 10.1002/1520-6629(199410)22:4<296::aid-jcop2290220403>3.0.co;2-t |
| **10** | United States (2012) | cross-sectional (observational) | middle school | 12-15 | 136  (47.1%) | Adolescent Minor Stress Inventory | Self-reported stress | boys (social) | Boys in Middle School | Blodgett Salafia, E., & Lemer, J. (2012). Associations Between Multiple Types of Stress and Disordered Eating Among Girls and Boys in Middle School. *Journal of Child & Family Studies,* 21(1), 148–157. DOI: 10.1007/s10826-011-9458-z |
| **11** | United States (2018) | clinical trial (experimental) | bars, clubs, businesses & organizations | M=24.6 | 296  (30%) | Structured Clinical Interview for DSM-IV | evaluate posttraumatic stress | men (social) | Emerging Adult Sexual Minority Men | Boroughs, M. S., Ehlinger, P. P., Batchelder, A. W., Safren, S. A., & O’Cleirigh, C. (2018). Posttraumatic Stress Symptoms and Emerging Adult Sexual Minority Men: Implications for Assessment and Treatment of Childhood Sexual Abuse. *Journal of Traumatic Stress,* 31(5), 665–675. DOI: 10.1002/jts.22335 |
| **12** | Netherlands (2008) | cohort (observational) | population | M=11.09 | 2127 (49.2%) | Stressful life events questionnaire | Number and severity of stressful life events encountered | boys (social) | early adolescent boys | Bouma, E. M. C., Ormel, J., Verhulst, F. C., Oldehinkel, A. J., EM, B., Ormel, J., FC, V., AJ, O., Bouma, E. M. C., Ormel, J., Verhulst, F. C., & Oldehinkel, A. J. (2008). Stressful life events and depressive problems in early adolescent boys and girls: The influence of parental depression, temperament and family environment. *Journal of Affective Disorders,* 105(1–3), 185–193. DOI: 10.1016/j.jad.2007.05.007 |
| **13** | England (2016) | laboratory (experimental) | university & community | M=21.83 | 25  (100%) | Stress and Arousal Checklist | feelings of stress and arousal | males (physiological) | young healthy males | Boyle, N. B., Lawton, C., Arkbåge, K., West, S. G., Thorell, L., Hofman, D., Weeks, A., Myrissa, K., Croden, F., & Dye, L. (2016). Stress responses to repeated exposure to a combined physical and social evaluative laboratory stressor in young healthy males. *Psychoneuroendocrinology,* 63, 119–127. DOI: 10.1016/j.psyneuen.2015.09.025 |
|  |  |  |  |  |  | Perceived Stress Scale | global measure of perceived stress |  |  |  |
| **14** | Brazil (2018) | laboratory (experimental) | children’s hospital | 11-12 | 80  (8%) | Escala de Stress Infantil (ESI) (Child Stress Scale) | Child stress | boys (social) | boys | Broering, C. V., de Souza, C. D., Kaszubowski, E., & Crepaldi, M. A. (2018). Effects of pre-surgical psychological preparations on stress and anxiety in boys and girls. *Acta Colombiana de Psicologia*, 21(1), 239–248. DOI: 10.14718/acp.2018.21.1.10 |
| **15** | United States (2015) | cross-sectional (observational) | social media & community venues | 16-24 | 260  (100%) | Concealment stress | concealment of sexual orientation during adolescence | males (physiological) | sexual minority male youth | Bruce, D., Harper, G. W., & Bauermeister, J. A. (2015). Minority stress, positive identity development, and depressive symptoms: Implications for resilience among sexual minority male youth. *Psychology of Sexual Orientation & Gender Diversity*, 2(3), 287–296. DOI: 10.1037/sgd0000128 |
| **16** | United States (2014) | cross-sectional (observational) | social media & community venues | 16-24 | 200  (100%) | Experience of sexual orientation stigma scale | minority stress | men (social) | young men who have sex with men | Bruce, D., Stall, R., Fata, A., & Campbell, R. T. (2014). Modeling minority stress effects on homelessness and health disparities among young men who have sex with men. *Journal of Urban Health,* 91(3), 568–580. DOI: 10.1007/s11524-014-9876-5 |
|  |  |  |  |  |  | Internalization of sexual orientation stigma |  |  |  |  |
| **17** | United States (1994) | cross-sectional (observational) | secure camp | 13-18 | 91  (100%) | PTSD diagnostic categories in DSM 111-R | symptom severity | males (physiological) | Male juvenile-offenders | Burton, D., Foy, D., Bwanausi, C., Johnson, J., & Moore, L. (1994). The relationship between traumatic exposure, family dysfunction, and posttraumatic stress symptoms in male juvenile-offenders. *Journal of Traumatic Stress,* 7(1), 83–93. DOI: 10.1007/bf02111914 |
| **18** | Canada (2019) | cross-sectional (observational) | online & local area | M=24.10 | 156  (100%) | Coping Inventory of Stressful Situations (CISS) | tendency to adopt coping strategies in response to daily stressful situations | males (physiological) | young male adults | Cantave, C. Y., Langevin, S., Marin, M.-F., Brendgen, M., Lupien, S., & Ouellet-Morin, I. (2019). Impact of maltreatment on depressive symptoms in young male adults: The mediating and moderating role of cortisol stress response and coping strategies. *Psychoneuroendocrinology,* 103, 41–48. DOI: 10.1016/j.psyneuen.2018.12.235 |
| **19** | United States (2013) | cross-sectional (observational) | population | 11-15 | 141  (39%) | Trauma Symptom Checklist for Children-PTS section | PTS symptoms | boys (social) | adolescent girls and boys | Cavanaugh, C. E. (2013). Brief report: The influence of posttraumatic stress on unprotected sex among sexually active adolescent girls and boys involved in the child welfare system of the United States. *Journal of Adolescence,* 36(5), 835–837. DOI: 10.1016/j.adolescence.2013.07.004 |
| **20** | Australia (2020) | cross-sectional (observational) | university & online forums | M=22.06 | 391  (100%) | Masculine Gender Role Discrepancy Stress Scale | masculinity discrepancy stress | men (social) | n/a | Cunningham, M. L., Rodgers, R. F., Lavender, J. M., Nagata, J. M., Frederick, D., Szabo, M., & Murray, S. B. (2020). “Big boys don’t cry”: Examining the indirect pathway of masculinity discrepancy stress and muscle dysmorphia symptomatology through dimensions of emotion dysregulation. *Body Image,* 34, 209–214. DOI: 10.1016/j.bodyim.2020.05.014 |
| **21** | Norway (2012) | cross-sectional (observational) | school | 17-19 | 2489 (45.7%) | Stressful life events questionnaire | number of events in past 12 months | boys (social) | 18-year-old Boys and Girls | Dalgard, F., Stern, R., Lien, L., & Hauser, S. (2012). Itch, Stress and Self-efficacy Among 18-year-old Boys and Girls: A Norwegian Population-based Cross-sectional Study. *Acta Dermato-Venereologica,* 92(5), 547–552. DOI: 10.2340/00015555-1309 |
| **22** | United States (2018) | cohort (observational) | urban area | 18-25 | 119  (100%) | Perceived Stress Scale | Perceived stress | males and men (physiological and social) | Ethnic minority emerging adult males | Desrosiers, A., Vine, V., & Kershaw, T. (2019). “RU Mad?”: Computerized text analysis of affect in social media relates to stress and substance use among ethnic minority emerging adult males. *Anxiety, Stress & Coping,* 32(1), 109–123. DOI: 10.1080/10615806.2018.1539964 |
| **23** | Iceland and Denmark (2010) | case-control  (observational) | population | 13-25 | 4,998 (16.4%) | Harvard Trauma Questionnaire | PTSD diagnosis and symptom severity | males and men (physiological and social) | men and women | Ditlevsen, D. N., Elklit, A., DN, D., & Elklit, A. (2010). The combined effect of gender and age on post traumatic stress disorder: do men and women show differences in the lifespan distribution of the disorder? *Annals of General Psychiatry,* 9, 1-12. DOI: 10.1186/1744-859X-9-32 |
| **24** | Canada (2012) | laboratory (experimental) | university | M=23.28 | 68  (36.8%) | Trier Inventory for the assessment of Chronic Stress (TICS) | Level of psychological stress experienced within previous month | men (social) | healthy young men and women | Duchesne, A., Tessera, E., Dedovic, K., Engert, V., & Pruessner, J. C. (2012). Effects of panel sex composition on the physiological stress responses to psychosocial stress in healthy young men and women. *Biological Psychology,* 89(1), 99–106. DOI: 10.1016/j.biopsycho.2011.09.009 |
| **25** | United States (2003) | cohort (observational) | county schools | 18-22 | 898  (100%) | Recent life events items | Social stress or strain | males (physiological) | young adult male | Eitle, D., & Turner, R. J. (2003). Stress exposure, race, and young adult male crime. *Sociological Quarterly,* 44(2), 243–269. DOI: 10.1525/tsq.2003.44.2.243 |
|  |  |  |  |  |  | Chronic stressors items |  |  |  |  |
|  |  |  |  |  |  | Lifetime major events items |  |  |  |  |
| **26** | United States (2004) | cohort (observational) | public schools | 18-23 | 838  (100%) | Preteen stress exposure | Events reported before age 12 | males (physiological) | male gang | Eitle, D., Gunkel, S., & Van Gundy, K. (2004). Cumulative exposure to stressful life events and male gang membership. *Journal of Criminal Justice*, 32(2), 95–111. DOI: 10.1016/j.jcrimjus.2003.12.001 |
|  |  |  |  |  |  | Family financial stress | family’s financial situation |  |  |  |
| **27** | Egypt and Saudi Arabia (2008) | cross-sectional (observational) | university | M=20.8 | 588  (100%) | Perceived Stress Scale | Sources of stress | males (physiological) | male medical students | El-Gilany, A.-H., Amr, M., & Hammad, S. (2008). Perceived stress among male medical students in Egypt and Saudi Arabia: effect of sociodemographic factors. *Annals of Saudi Medicine*, 28(6), 442–448. DOI: 10.5144/0256-4947.2008.442 |
| **28** | United States (2019) | cohort (observational) | social media, venues & organizations | M=22.85 | 109  (100%) | Perceived  Stress Scale | General stress | assigned male at birth (physiological) | Young male same-sex couples | Feinstein, B. A., McConnell, E., Dyar, C., Mustanski, B., & Newcomb, M. E. (2019). The influence of stress on depression and substance use problems among young male same-sex couples: Relationship functioning as an underlying mechanism. *Clinical Psychological Science*, 7(5), 928–940. DOI: 10.1177/2167702619842561 |
| **29** | United States (2018) | cohort (observational) | population | M=23.06 | 306  (100%) | Perceived  Stress Scale | General stress | assigned male at birth (physiological) | Young male same-sex couples | Feinstein, B. A., McConnell, E. D. C., Mustanski, B., & Newcomb, M. E. (2018). Minority stress and relationships functioning among young male same-sex couples: An examination of actor-partner interdependence model. *Journal of Consulting & Clinical Psychology*, 86(5), 416–426. DOI: 10.1037/ccp0000296 |
| **30** | United States (2006) | cohort (observational) | elementary schools | M=16.1 | 125  (100%) | Life Events Questionnaire Adolescent Versions (LEQ-C & LEQ-A) | experience of negative life events | males (physiological) | urban male adolescents | Fishbein, D. H., Herman-Stahl, M., Eldreth, D., Paschall, M. J., Hyde, C., Hubal, R., Hubbard, S., Williams, J., & Ialongo, N. (2006). Mediators of the stress-substance-use relationship in urban male adolescents. *Prevention Science*, 7(2), 113–126. DOI: 10.1007/s11121-006-0027-4 |
|  |  |  |  |  |  | Adolescent Perceived Events Scale (APES) |  |  |  |  |
|  |  |  |  |  |  | Adolescent-Family Inventory of Life Events and Changes (A-FILE) |  |  |  |  |
| **31** | Germany (2002) | case-control (observational) | parental support group | M=11.95 | 127  (61.4%) | Questionnaire on Resources and Stress | Parental stress | males and boys (physiological and social) | boys with Fragile X syndrome and Spinal Muscular Atrophy | Gontard, A. von, Backes, M., Laufersweiler-Plass, C., Wendland, C., & al, et. (2002). Psychopathology and familial stress--Comparison of boys with Fragile X syndrome and Spinal Muscular Atrophy. *Journal of Child Psychology & Psychiatry* & *Allied Disciplines*, 43(7), 949–957. DOI: 10.1111/1469-7610.00098 |
| **32** | Japan (2004) | cross-sectional (observational) | universities | M=18.4 | 916  (38.5%) | Stress-coping skills items | Active and passive coping | males (physiological) | n/a | Hirokawa, K., Yagi, A., & Miyata, Y. (2004). An Examination of Masculinity-Feminity Traits and Their Relationships to Communication Skills and Stress-Coping Skills. *Social Behavior & Personality*, 32(8), 731–740. DOI: 10.2224/sbp.2004.32.8.731 |
| **33** | United States (2008) | cross-sectional (observational) | population | M=13.9 | 37  (100%) | Perceived  Stress Scale | subjects’ perception of stress in their lives over the last month | males and boys (physiological and social) | 8-to 18-Year-Old Boys | Holmes, M. E., Eisenmann, J. C., Ekkekakis, P., & Gentile, D. (2008). Physical Activity, Stress, and Metabolic Risk Score in 8-to 18-Year-Old Boys. *Journal of Physical Activity & Health*, 5(2), 294–307. DOI: 10.1123/jpah.5.2.294 |
| **34** | United States (2018) | laboratory (experimental) | correctional /treatment facility | 14–18 | 28  (100%) | Life Stress Interview (LSI) | chronic stressors across domains in past year | males (physiological) | incarcerated male adolescents | Johnson, M., Vitacco, M. J., & Shirtcliff, E. A. (2018). Callous-unemotional traits and early life stress predict treatment effects on stress and sex hormone functioning in incarcerated male adolescents. *Stress*, 21(2), 110–118. DOI: 10.1080/10253890.2017.1414799 |
| **35** | Australia (2020) | cohort (observational) | population | M=21 | 429  (45%) | Depression Anxiety Stress Scale  (DASS-21) | Depression, anxiety, and stress symptoms | males (physiological) | young adult males | Kaur, S., Christian, H., Cooper, M. N., Francis, J., Allen, K., & Trapp, G. (2020). Consumption of energy drinks is associated with depression, anxiety, and stress in young adult males: Evidence from a longitudinal cohort study. *Depression & Anxiety*, (1091-4269), 37(11), 1089–1098. DOI: 10.1002/da.23090 |
| **36** | Kosovo (2020) | cross-sectional (observational) | university | M=20 | 445  (100%) | Masculine Gender Role Stress Scale | masculine gender role stress | males (physiological) | Male Students | Kelmendi, K. (2020). Exploring Masculine Gender Role Stress and Intimate Partner Violence Among Male Students in Kosovo. *Psychology of Men & Masculinities,* 21(3), 479–489. DOI: 10.1037/men0000229 |
| **37** | India (2012) | cross-sectional (observational) | high school | 13-15 | 120  (50%) | Scale for Assessing Academic Stress (SASS) | Assessing presence or absence of academic stress | boys (social) | High School Girl and Boy Students | Khanehkeshi, A., & Basavarajappa. (2012). A Comparative Study of the Academic Stress and Depression among High School Girl and Boy Students. *Journal on Educational Psychology*, 6(1), 11–20. |
| **38** | Korea (2017) | cross-sectional (observational) | university | 23-25 | 281  (89.3%) | Life Stress Scale for College Students | IV on problem drinking | males (physiological) | Male University Students | Kim, E. J. (2017). Problem Drinking Behavior and Related Factors in Male University Students According to Alcohol Use Disorder, Drinking Motivation, Coping Style and Stress. *International Information Institute (Tokyo)*. Information, 20(9B), 7051–7058. |
| **39** | United States (2020) | cohort (observational) | venues and social media | 16-24 | 448  (100%) | stressful life events questionnaire | number and severity of stressful events experienced | men (social) | Black and Latino Young Men Who Have Sex with Men | Kipke, M. D., Kubicek, K., Akinyemi, I. C., Hawkins, W., Belzer, M., Bhandari, S., & Bray, B. (2020). The Healthy Young Men’s Cohort: Health, Stress, and Risk Profile of Black and Latino Young Men Who Have Sex with Men (YMSM). *Journal of Urban Health*, 97(5), 653–667. DOI: 10.1007/s11524-019-00398-6 |
| **40** | United States (2011) | cross-sectional (observational) | community | M=20.1 | 79  (100%) | Racist Hassles Questionnaire | Young adult contextual stressors | men (social) | young adult men | Kogan, S. M., Brody, G. H., Chen, Y.-F., & DiClemente, R. J. (2011). Self-regulatory problems mediate the association of contextual stressors and unprotected intercourse among rural, African American, young adult men. *Journal of Health Psychology*, 16(1), 50–57. DOI: 10.1177/1359105310367831 |
|  |  |  |  |  |  | Community Resources and Problems Measure |  |  |  |  |
| **41** | United States (2017) | laboratory (experimental) | university | M=18.8 | 285  (100%) | Perceived Social Stress Test | evaluate life stressors participant experienced over prior month | males and men (physiological and social) | n/a | Kramer MA, B. L., Himmelstein PhD, M. S., & Springer PhD, MPH, K. W. (2017). Getting to the Heart of Masculinity Stressors: Masculinity Threats Induce Pronounced Vagal Withdrawal During a Speaking Task. *Annals of Behavioral Medicine,* 51(6), 846–855. DOI: 10.1007/s12160-017-9907-z |
| **42** | United States (2002) | cross-sectional (observational) | university | M=20.27 | 214  (51.9%) | Perceived Stress Scale | stress and worry over the inability to handle life demands | men (social) | college women and men | Lee, R. M., Keough, K. A., & Sexton, J. D. (2002). Social connectedness, social appraisal, and perceived stress in college women and men. *Journal of Counseling & Development*, 80(3), 355–361. DOI: 10.1002/j.1556-6678.2002.tb00200.x |
| **43** | China (2020) | cross-sectional (observational) | college | M=22.07 | 59  (100%) | Hindrance and Challenge Stress Scale | Academic stress | males (physiological) | male college students | Lin, L., Zhang, J., Wang, P., Bai, X., Sun, X., & Zhang, L. (2020). Perceived control moderates the impact of academic stress on the attention process of working memory in male college students. *Stress*, 23(3), 256–264. DOI: 10.1080/10253890.2019.1669557 |
| **44** | Sweden (2017) | cross-sectional (observational) | schools | 14-16 | 413  (30.6%) | activation and pressure stress (PAS) scale | Perceived stress | boys (social) | mid-adolescent girls and boys | Lindfors, P., Folkesson Hellstadius, L., & Östberg, V. (2017). Perceived stress, recurrent pain, and aggregate salivary cortisol measures in mid-adolescent girls and boys. *Scandinavian Journal of Psychology*, 58(1), 36–42. DOI: 10.1111/sjop.12347 |
| **45** | Germany (2010) | laboratory (experimental) | single-gender boys’ schools | 12-14 | 98  (100%) | Positive and Negative Affect Schedule | Distress and eustress | boys (social) | adolescent boys | Maass, A., Lohaus, A., & Wolf, O. T. (2010). Media and stress in adolescent boys in Germany: psychophysiological effects of violent and non-violent programs and video games. *Journal of Children & Media*, 4(1), 18–38. DOI: 10.1080/17482790903407259 |
| **46** | Canada (1996) | cross-sectional (observational) | private college | M=21 | 119  (88.2%) | Masculine Gender Role Stress Scale | masculine gender role stress | males (physiological) | n/a | McCreary, D. R., Wong, F. Y., Wiener, W., Carpenter, K. M., Engle, A., & Nelson, P. (1996). The Relationship Between Masculine Gender Role Stress and Psychological Adjustment: A Question of Construct Validity? *Sex Roles*, 34(7), 507. DOI: 10.1007/BF01545029 |
| **47** | United States (2013) | cross-sectional (observational) | university | M=22.29 | 419  (100%) | Masculine Gender Role Stress Scale | experience stress in situations that challenge traditionally defined cultural standards of masculinity | men (social) | College Men | McDermott, R. C., & Lopez, F. G. (2013). College Men’s Intimate Partner Violence Attitudes: Contributions of Adult Attachment and Gender Role Stress. *Journal of Counseling Psychology*, 60(1), 127–136. DOI: 10.1037/a0030353 |
| **48** | Iran (2008) | cross-sectional (observational) | high school | M=17.5 | 154  (100%) | Persian version of the Perceived Stress Scale | Perception of life event in past month as stressful | males (physiological) | Iranian male high school students | Moeini, B., Shafii, F., Hidarnia, A., Babaii, G. R., Birashk, B., & Allahverdipour, H. (2008). Perceived Stress, Self-efficacy and its Relations to Psychological Well-being Status in Iranian Male High School Students. *Social Behavior* & *Personality*, 36(2), 257–266. DOI: 10.2224/sbp.2008.36.2.257 |
| **49** | Australia (2013) | cohort (observational) | school | M=15.36 | 298 (46%) | Adolescent Stress Questionnaire (ASQ) | perceived stress associated of life domains during adolescence | males (physiological) | adolescent females and males | Murray, K., Rieger, E., & Byrne, D. (2013). A longitudinal investigation of the mediating role of self-esteem and body importance in the relationship between stress and body dissatisfaction in adolescent females and males. *Body Image*, 10(4), 544–551. DOI: 10.1016/j.bodyim.2013.07.011 |
| **50** | Australia (2015) | cross-sectional (observational) | non-government coeducational high  schools | 12-16 | 515  (50.3%) | Adolescent Stress Questionnaire (ASQ) | Exposure and appraisal of stressors over 5 years | males (physiological) | Female and Male Adolescents | Murray, K., Rieger, E., & Byrne, D. (2015). The Relationship Between Stress and Body Satisfaction in Female and Male Adolescents. *Stress & Health*, 31(1), 13–23. DOI: 10.1002/smi.2516 |
| **51** | Australia (2016) | laboratory (experimental) | university | 18-25 | 111  (40.5%) | Peer stress | IV on body dissatisfaction | males (physiological) | Female and Male Young Adults | Murray, K., Rieger, E., & Byrne, D. (2016). The Effect of Peer Stress on Body Dissatisfaction in Female and Male Young Adults. *Journal of Experimental Psychopathology*, 7(2), 261–276. DOI: 10.5127/jep.046514 |
|  |  |  |  |  |  | Physical Appearance State and Trait Anxiety Scale (PASTAS; | Weight-related distress |  |  |  |
| **52** | Australia (2008) | cross-sectional (observational) | gymnasium foyers, lecture theatres | M=24.38 | 129  (100%) | Masculine Gender Role Stress (MGRS) | perceptions of scenarios as stressful | men (social) | n/a | Mussap, A. J. (2008). Masculine Gender Role Stress and the Pursuit of Muscularity. *International Journal of Men’s Health*, 7(1), 72-79,81-82,84-89. DOI:10.3149/jmh.0701.72 |
| **53** | Denmark (2020) | cross-sectional (observational) | population | 19 (median) | 1362 (100%) | Social Readjustment scale | Experience of stressful life event in previous 3 months | men (social) | young men | Nordkap, L., Priskorn, L., Brauner, E. V, Marie Hansen, A., Kirstine Bang, A., Holmboe, S. A., Winge, S. B., Egeberg Palme, D. L., Morup, N., Erik Skakkebaek, N., Kold Jensen, T., & Jorgensen, N. (2020). Impact of psychological stress measured in three different scales on testis function: A cross-sectional study of 1362 young men. *Andrology*, 8(6), 1674–1686. DOI: 10.1111/andr.12835 |
|  |  |  |  |  |  | Danish version of Perceived Stress Scale | perceived stress during the last four weeks |  |  |  |
| **54** | England and Scotland (2005) | laboratory (experimental) | university | M=20.41 | 297  (51.1%) | Appraisals of life events(ALE) scale | Appraisals of threat, challenge and loss | males (physiological) | n/a | O’Connor, D. B., O., Ferguson, E., & O’Connor, R. C. (2005). Intentions to use hormonal male contraception: the role of message framing, attitudes and stress appraisals. *British Journal of Psychology,* 96(3), 351–369. DOI: 10.1348/000712605X49114 |
| **55** | Sweden (2015) | cross-sectional (observational) | schools | 14-16 | 545  (43.4%) | Perceived Stress | perceived stress reactions and general demands | boys (social) | Mid-Adolescent Girls and Boys | Östberg, V., Almquist, Y. B., Folkesson, L., Låftman, S. B., Modin, B., & Lindfors, P. (2015). The Complexity of Stress in Mid-Adolescent Girls and Boys: Findings from the Multiple Methods School Stress and Support Study. *Child Indicators Research*, 8(2), 403–423. DOI: 10.1007/s12187-014-9245-7 |
| **56** | Sweden (2018) | cross-sectional (observational) | compulsory schools | 14-16 | 411  (40.6%) | Pressure-Activation Stress (PAS) Scale | Perceived stress | boys (social) | Mid-Adolescent Girls and Boys | Östberg, V., Plenty, S., Låftman, S. B., Modin, B., & Lindfors, P. (2018). School Demands and Coping Resources-Associations with Multiple Measures of Stress in Mid-Adolescent Girls and Boys. *International Journal of Environmental Research & Public Health*, 15(10). DOI: 10.3390/ijerph15102143 |
| **57** | United States (2003) | clinical trial  (experimental) | corrections facility | 13-18 | 43  (100%) | Post Traumatic Stress Disorder Reaction Index (PTSD-RI) | Posttraumatic stress reactions to traumatic events | males (physiological) | incarcerated male juveniles | Ovaert, L. B., Cashel, M. L., & Sewell, K. W. (2003). Structured group therapy for posttraumatic stress disorder in incarcerated male juveniles. *American Journal of Orthopsychiatry*, 73(3), 296–303. DOI: 10.1037/0002-9432.73.2.294 |
|  |  |  |  |  |  | Children’s PTSD Inventory | diagnostic |  |  |  |
| **58** | India (2016) | cross-sectional (observational) | higher secondary school | 16-18 | 203  (50.7%) | Hindi adaptation of Student Academic Stress Scale | academic stress | boys (social) | Boys and girls students | Pandey, D. (2016). Self-concept and academic stress among boys and girls students. *Indian Journal of Health & Wellbeing,* 7(5), 540-542. |
| **59** | United States (1998) | cross-sectional (observational) | city | 14-19 | 188  (100%) | Multi-item scales including: financial strain, depression, neighbourhood problems | Family stress and conflict | males (physiological) | African American male adolescents’ | Paschall, M. J., & Hubbard, M. L. (1998). Effects of neighborhood and family stressors on African American male adolescents’ self-worth and propensity for violent behavior. *Journal of Consulting & Clinical Psychology*, 66(5), 825–831. DOI: 10.1037/0022-006X.66.5.825 |
| **60** | Canada (2003) | cohort (observational) | university | M=24.3 | 40  (100%) | English version of Trier Inventory for the Assessment of  Chronic Stress (TICS) | Chronic stress | males (physiological) | healthy young men | Pruessner, M., DH, H., JC, P., SJ, L., Pruessner, M., Hellhammer, D. H., Pruessner, J. C., & Lupien, S. J. (2003). Self-reported depressive symptoms and stress levels in healthy young men: associations with the cortisol response to awakening. *Psychosomatic Medicine*, 65(1), 92–99. DOI: 10.1097/01.psy.0000040950.22044.10 |
|  |  |  |  |  |  | 10-point  rating scale | Acute stress |  |  |  |
| **61** | United States (2018) | cohort (observational) | university | 18-22 | 157  (50.3%) | Perceived Stress Scale | Perception of life events as stressful over the past month | men (social) | Women and Men | Raisanen, J. C., Chadwick, S. B., Michalak, N., & van Anders, S. M. (2018). Average Associations Between Sexual Desire, Testosterone, and Stress in Women and Men Over Time. *Archives of Sexual Behavior*, 47(6), 1613–1631. DOI: 10.1007/s10508-018-1231-6 |
| **62** | United States (2015) | cross-sectional (observational) | middle and  high schools | 11-18 | 589  (100%) | 5 Likert-type questions | Gender role discrepancy and discrepancy stress | males (physiological) | adolescent boys | Reidy, D. E., Smith-Darden, J. P., Cortina, K. S., Kernsmith, R. M., & Kernsmith, P. D. (2015). Masculine discrepancy stress, teen dating violence, and sexual violence perpetration among adolescent boys. *The Journal of Adolescent Health*, 56(6), 619–624. DOI: 10.1016/j.jadohealth.2015.02.009 |
| **63** | United States (2018) | cross-sectional (observational) | middle and high schools | 11-16 | 592  (100%) | Masculine Discrepancy Stress Scale | Gender Role discrepancy and masculine discrepancy stress | males (physiological) | Adolescent Boys | Reidy, D. E., Smith-Darden, J. P., Vivolo-Kantor, A. M., Malone, C. A., & Kernsmith, P. D. (2018). Masculine Discrepancy Stress and Psychosocial Maladjustment: Implications for Behavioral and Mental Health of Adolescent Boys. *Psychology of Men & Masculinity*, 19(4). DOI: 10.1037/men0000132 |
| **64** | United States (1996) | cross-sectional (observational) | recreational and  social services | 14-19 | 136  (100%) | anxiety, depression, and phobic anxiety subscales of Derogatis’ Symptom Checklist-90 (SCL-90) | Emotional distress | males (physiological) | gay and bisexual male adolescents of predominantly Black and Hispanic background | Rosario, M., Rotheram-Borus, M. J., & Reid, H. (1996). Gay-related stress and its correlates among gay and bisexual male adolescents of predominantly Black and Hispanic background. *Journal of Community Psychology*, 24(2), 136–159. DOI: 10.1002/(SICI)1520-6629(199604)24:2<136::AID-JCOP5>3.0.CO;2-X |
|  |  |  |  |  |  | 7-item scale | Gay-related stressful life events |  |  |  |
|  |  |  |  |  |  | Life Events Checklist | Non-guy-related stressful life events |  |  |  |
| **65** | Switzerland (2018) | cohort (observational) | military service recruitment centre | M=21.3 | 5308 (100%) | Post-traumatic Diagnostic Scale | Exposure to stressful potentially traumatic incidents | men (social) | young men | Rougemont-Bucking, A., Grazioli, V. S. V. S., Marmet, S., Daeppen, J.-B., Lemoine, M. M., Gmel, G., Studer, J., Rougemont-Bücking, A., Grazioli, V. S. V. S., Marmet, S., Daeppen, J.-B., Lemoine, M. M., Gmel, G., & Studer, J. (2018). Non-medical use of prescription drugs by young men: impact of potentially traumatic events and of social-environmental stressors. *European Journal of Psychotraumatology*, 9(1), 1468706. DOI: 10.1080/20008198.2018.1468706 |
|  |  |  |  |  |  | 6 social-environmental stressors | assessment of chronic social-environmental stressors |  |  |  |
| **66** | Switzerland (2017) | cohort (observational) | military service recruitment centre | M=21.3 | 5308 (100%) | post-traumatic diagnostic scale | Exposure to external stress factors | men (social) | young men | Rougemont-Bücking, A., Grazioli, V. S., Daeppen, J. B., Gmel, G., & Studer, J. (2017). Family-related stress versus external stressors: Differential impacts on alcohol and illicit drug use in young men. *European Addiction Research*, 23(6), 284-297. DOI: 10.1159/000485031 |
|  |  |  |  |  |  | Life Event Checklist |  |  |  |  |
|  |  |  |  |  |  | Trauma History Questionnaire |  |  |  |  |
|  |  |  |  |  |  | European School Survey Project  on Alcohol and Drugs (ESPAD) | family-related stress factors |  |  |  |
|  |  |  |  |  |  | family history section from the Addiction Severity Index |  |  |  |  |
| **67** | India (2015) | cross-sectional (observational) | English medium schools | 12-15 | 1153 (100%) | Manipal stress questionnaire (MSQ) | stressors and stress tolerance level | boys  (social) | adolescent boys | Roy, K., Kamath, V. G., Kamath, A., Alex, J., & Hegde, A. (2017). Prevalence of stress and stress tolerance levels among adolescent boys - a district level cross sectional study in South India. *International Journal of Adolescent Medicine* & *Health*, 29(4), 1--7. DOI: 10.1515/ijamh-2015-0054 |
| **68** | Russia (1998) | cross-sectional (observational) | male juvenile correction | 15-17 | 15  (100%) | Revised Impact of Event Scale | intrusive and avoidance symptoms related to the traumatic event | males (physiological) | Juvenile male rape victims | Ruchkin, V. V, Eisemann, M., & Hägglöf, B. (1998). Juvenile male rape victims: is the level of post-traumatic stress related to personality and parenting? *Child Abuse & Neglect*, 22(9), 889–899. DOI: 10.1016/S0145-2134(98)00064-7 |
|  |  |  |  |  |  | Child Self-report Post-traumatic Stress Reaction Index | post-traumatic stress reactions to traumatic events |  |  |  |
|  |  |  |  |  |  | Hospital Anxiety and Depression Scale | detecting states of depression and anxiety |  |  |  |
|  |  |  |  |  |  | Temperament and Character Inventory | dimensions of temperament and personality |  |  |  |
|  |  |  |  |  |  | EMBU (own memories of parental rearing) | perceived parental rearing behavior |  |  |  |
| **69** | India (2010) | cross-sectional (observational) | colleges | 17-22 | 405  (100%) | Depression, Anxiety, and Stress Scale 21 (DASS-21) | negative emotional states of depression, anxiety, and stress | males (physiological) | young male adults | Sahoo, S., CR, K., Sahoo BA, MBBS, DPM, MD, S., Khess MD, C. R. J., Sahoo, S., & CR, K. (2010). Prevalence of depression, anxiety, and stress among young male adults in India: a dimensional and categorical diagnoses-based study. *Journal of Nervous & Mental Disease*, 198(12), 901–904. DOI: 10.1097/NMD.0b013e3181fe75dc |
| **70** | Sweden (2018) | cross-sectional (observational) | elementary schools | 14-16 | 392  (40.8%) | 1-item question | Global stress | boys  (social) | Mid-Adolescent Girls and Boys | Sahu, L., Jha, M., & Pandey, D. (2016). Self-concept and academic stress among Ostberg, V., Laftman, S. B., Modin, B., Lindfors, P., Östberg, V., Låftman, S. B., Modin, B., Lindfors, P., Ostberg, V., Laftman, S. B., Modin, B., Lindfors, P., Östberg, V., Låftman, S. B., Modin, B., & Lindfors, P. (2018). Bullying as a Stressor in Mid-Adolescent Girls and Boys-Associations with Perceived Stress, Recurrent Pain, and Salivary Cortisol. *International Journal of Environmental Research* & *Public Health*, 15(2), 364. DOI: 10.3390/ijerph15020364 |
|  |  |  |  |  |  | Pressure and Activation Stress (PAS) Scale | Perceived stress |  |  |  |
| **71** | United States (2012) | cohort (observational) | MST provider agencies | 12-18 | 120  (100%) | Life Events Checklist (LEC) | recent episodic stressors | males (physiological) | adolescent males | Schechter, J. C., Brennan, P. A., Cunningham, P. B., Foster, S. L., & Whitmore, E. (2012). Stress, cortisol, and externalizing behavior in adolescent males: an examination in the context of multisystemic therapy. *Journal of Abnormal Child Psychology*, 40(6), 913–922. DOI: 10.1007/s10802-012-9612-0 |
|  |  |  |  |  |  | Urban Hassles Scale | daily hassles encountered |  |  |  |
|  |  |  |  |  |  | Lifetime stress questionnaire | Experience of major negative life events |  |  |  |
| **72** | United States (2015) | cohort (observational) | foster care | 18-19 | 74  (100%) | Black Male Experiences Measure (BMEM) | Experiences and perceptions of Black males | males (physiological) | Young Black Males | Scott Jr., L. D., McCoy, H., Scott Jr, L. D., McCoy, H., Scott, L. D., & McCoy, H. (2015). Negative Social Contextual Stressors and Somatic Symptoms Among Young Black Males: An Exploratory Study. *Journal of Human Behavior in the Social Environment*, 25(8), 885–896. DOI: 10.1080/10911359.2015.1039155 |
| **73** | United States (2013) | cross-sectional (observational) | barber shops and recreational facilities | M=23.4 | 80  (100%) | City Stress Inventory (CSI) | neighborhood disorder and exposure to neighborhood violence | men (social) | young adult African American men | Seth, P., Murray, C. C., Braxton, N. D., & Diclemente, R. J. (2013). The concrete jungle: city stress and substance abuse among young adult African American men. *Journal of Urban Health*, 90(2), 307–313. DOI: 10.1007/s11524-012-9716-4 |
| **74** | United States (2014) | cross-sectional (observational) | secure confinement facility | 14-19 | 238  (100%) | UCLA PTSD Index for DSM-IV (Adolescent Version, Revision 1) | PTSD symptoms | males (physiological) | Incarcerated Boys | Sharf, A., Kimonis, E. R., & Howard, A. (2014). Negative Life Events and Posttraumatic Stress Disorder among Incarcerated Boys with Callous-Unemotional Traits. *Journal of Psychopathology & Behavioral Assessment*, 36(3), 401–414. DOI: 10.1007/s10862-013-9404-z |
| **75** | Germany (2017) | laboratory (experimental) | university | M=21.90 | 55  (100%) | German state version of the Positive and Negative Affect Schedule (PANAS) | emotional stress reactivity | males (physiological) | young healthy men | Singer, N., Sommer, M., Döhnel, K., Zänkert, S., Wüst, S., & Kudielka, B. M. (2017). Acute psychosocial stress and everyday moral decision-making in young healthy men: The impact of cortisol. *Hormones & Behavior*, 93, 72–81. DOI: 10.1016/j.yhbeh.2017.05.002 |
|  |  |  |  |  |  | German version of the Primary Appraisal Secondary Appraisal (PASA) questionnaire | anticipatory and retrospective cognitive stress appraisal |  |  |  |
| **76** | United States (2014) | cross-sectional (observational) | juvenile detention centers | 12-16 | 66  (100%) | UCLA PTSD Index for DSM–IV | trauma and PTSD symptoms | males and boys (physiological and social) | Male Juvenile Offenders | Stimmel, M. A., Cruise, K. R., Ford, J. D., & Weiss, R. A. (2014). Trauma Exposure, Posttraumatic Stress Disorder Symptomatology, and Aggression in Male Juvenile Offenders. *Psychological Trauma: Theory, Research, Practice & Policy*, 6(2), 184–191. DOI: 10.1037/a0032509 |
| **77** | United States (2019) | cohort (observational) | metropolitan areas | M=24.86 | 1817 (100%) | experienced homophobia | Minority Stress | men (social) | Young Black Men who have Sex with Men | Storholm, E. D., Huang, W., Siconolfi, D. E., Pollack, L. M., Carrico, A. W., Vincent, W., Rebchook, G. M., Huebner, D. M., Wagner, G. J., & Kegeles, S. M. (2019). Sources of Resilience as Mediators of the Effect of Minority Stress on Stimulant Use and Sexual Risk Behavior Among Young Black Men who have Sex with Men. *AIDS & Behavior*, 23(12), 3384–3395. DOI: 10.1007/s10461-019-02572-y |
|  |  |  |  |  |  | experienced racism |  |  |  |  |
|  |  |  |  |  |  | internalized homophobia |  |  |  |  |
| **78** | Netherlands (1997) | laboratory (experimental) | university | M=23 | 36  (100%) | Everyday  Problem Checklist (Alledaagse  Problemen Lijst) | indicator of chronic stress | males (physiological) | male students | Straatman, I., Hanson, E. K. S., Endenburg, N., & Mol, J. A. (1997). The influence of a dog on male students during a stressor. *Anthrozoos*, 10(4), 191–197. DOI: 10.2752/089279397787001012 |
| **79** | United States (2015) | cross-sectional (observational) | large urban location | 19-24 | 1729 (100%) | Masculine Gender Role Stress Scale | men’s experience of stress associated with events related to the male gender role | males and men (physiological and social) | n/a | Swartout, K. M., Parrott, D. J., Cohn, A. M., Hagman, B. T., & Gallagher, K. E. (2015). Development of the Abbreviated Masculine Gender Role Stress Scale. *Psychological Assessment*, 27(2), 489–500. DOI: 10.1037/a0038443 |
| **80** | Japan (2011) | cross-sectional (observational) | university | M=24.8 | 37  (100%) | Japanese Perceived Stress Scale (PSS) | Perceived stress | males (physiological) | asymptomatic young men | Tanaka, G., Kato, Y., Matsumura, K., Horiguchi, M., Ogasawara, H., & Sawada, Y. (2011). The association between chronic psychosocial stress, allostatic load, and vascular health in asymptomatic young men: A pilot study using a novel finger arterial stiffness index. *Japanese Psychological Research*, 53(2), 140–154. DOI: 10.1111/j.1468-5884.2011.00461.x |
|  |  |  |  |  |  | Japanese version of Sense of Coherence 13 | positive psychological traits that moderate coping with a stressful condition |  |  |  |
| **81** | United States (2012) | cross-sectional (observational) | juvenile facility | 14-17 | 355  (100%) | Stressful Life Events scale | histories of trauma and victimization | males (physiological) | Male Juvenile Offenders | Tatar, J. R., Cauffman, E., Kimonis, E. R., & Skeem, J. L. (2012). Victimization History and Posttraumatic Stress: An Analysis of Psychopathy Variants in Male Juvenile Offenders. *Journal of Child & Adolescent Trauma*, 5(2), 102–113. DOI: 10.1080/19361521.2012.671794 |
|  |  |  |  |  |  | Schedule for Affective Disorders and Schizophrenia for School-Age Children–Present and Lifetime version (K-SADS-PL) | Symptoms of PTSD |  |  |  |
| **82** | Brazil (2020) | cross-sectional (observational) | compulsory military service | M=18 | 236  (100%) | Perceived Stress Scale (Brazilian Portuguese version) | lack of control and negative affective reactions | males (physiological) | young male military recruits | Tonon, A. C., Carissimi, A., Schimitt, R. L., de Lima, L. S., Pereira, F. dos S., & Hidalgo, M. P. (2020). How do stress, sleep quality, and chronotype associate with clinically significant depressive symptoms? A study of young male military recruits in compulsory service. *Brazilian Journal of Psychiatry*, 42(1), 54–62. DOI: 10.1590/1516-4446-2018-0286 |
| **83** | India (2019) | cross-sectional (observational) | population | 18-25 | 30  (50%) | Perceived Stress Scale | Perceived stress | males (physiological) | adolescent males and females | Udayakumar, K. P., Sureshkumar, P., & Kuppusamy, T. S. (2019). Assessment of stress and cognition among adolescent males and females. *National Journal of Physiology, Pharmacy & Pharmacology*, 9(1), 43–47. DOI: 10.5455/njppp.2019.9.1032913112018 |
| **84** | United States (2017) | laboratory (experimental) | campus, neighborhoods, & online classifieds & forums | 15-17 | 22  (13.3%) | ecological momentary assessment (EMA) | naturally occurring daily stress | boys  (social) | adolescent boys | Uy, J. P., & Galvan, A. (2017). Acute stress increases risky decisions and dampens prefrontal activation among adolescent boys. *Neuroimage*, 146, 679–689. DOI: 10.1016/j.neuroimage.2016.08.067 |
| **85** | Netherlands (2005) | cross-sectional (observational) | university | 22.37 | 2023 (40.6%) | MGRS scale | masculine gender role stress | males and men (physiological and social) | n/a | van Well, S., Kolk, A. M., & Arrindell, W. A. (2005). Cross-cultural validity of the masculine and Feminine Gender Role Stress scales. *Journal of Personality Assessment*, 84(3), 271–278. DOI: 10.1207/s15327752jpa8403_06 |
| **86** | Mexico (2019) | cross-sectional (observational) | public high school | M=15.90 | 1417  (43%) | Adolescents Life Events Questionnaire | Occurrence of stressful life events in last six months | males (physiological) | Mexican adolescents: Male vs. female | Veytia-Lopez, M., Calvete, E., Sanchez-Alvarez, N., & Guadarrama-Guadarrama, R. (2019). Relationship between stressful life events and emotional intelligence in Mexican adolescents: Male vs. female comparative study. *Salud Mental*, 42(6), 261–268. DOI: 10.17711/SM.0185-3325.2019.034 |
| **87** | United States (2018) | clinical trial (experimental) | collegiate soccer | 18-22 | 18  (100%) | Perceived Stress Scale (PSS) | beliefs of how stressful certain parts are in their life | males and men (physiological and social) | Male Collegiate Soccer Players | Vidic, Z., Martin, M. St., & Oxhandler, R. (2018). Mindfulness Meditation Intervention with Male Collegiate Soccer Players: Effect on Stress and Various Aspects of Life. *Sport Journal*, 1. |
| **88** | United States (2017) | cross-sectional (observational) | neighborhoods | 23 (median) | 618  (100%) | 20 items Life stress | frequency of exposure to stressful events | born male and men  (physiological and social) | young Black men who have sex with men | Voisin, D. R., Hotton, A. L., Schneider, J. A., & Team, T. Uc. S. (2017). The relationship between life stressors and drug and sexual behaviors among a population-based sample of young Black men who have sex with men in Chicago. *AIDS Care*, 29(5), 545–551. DOI: 10.1080/09540121.2016.1224303 |
| **89** | Lebanon (2018) | cohort (observational) | community & social media | M=23.9 | 226  (100%) | Multiple Discriminations Scale | Sexual minority-related discrimination | biologically male and male-identified (physiological and social) | Young Men Who Have Sex with Men | Wagner, G. J., Ghosh-Dastidar, B., El Khoury, C., Ghanem, C. A., Balan, E., Kegeles, S., Mutchler, M. G., & Mokhbat, J. (2019). Major Depression Among Young Men Who Have Sex with Men in Beirut, and Its Association with Structural and Sexual Minority-Related Stressors, and Social Support. *Sexuality Research & Social Policy*, 16(4), 513–520. DOI: 10.1007/s13178-018-0352-y |
|  |  |  |  |  |  | Comfort with sexual identity |  |  |  |  |
| **90** | United States (2014) | cohort (observational) | school | 11-14 | 341  (100%) | stress and coping measure | Exposure to stress | males (physiological) | Urban Adolescent Males | Williams, J., Aiyer, S., Durkee, M., & Tolan, P. (2014). The Protective Role of Ethnic Identity for Urban Adolescent Males Facing Multiple Stressors. *Journal of Youth & Adolescence*, 43(10), 1728–1741. DOI: 10.1007/s10964-013-0071-x |
| **91** | United States (2014) | cross-sectional (observational) | public university | M=24.98 | 160  (100%) | Perceived Discrimination subscale of  the Acculturative Stress Scale for International Students | Perceived racial discrimination | males (physiological) | Male Asian international students | Wong, Y. J., Tsai, P.-C., Liu, T., Zhu, Q., & Wei, M. (2014). Male Asian international students’ perceived racial discrimination, masculine identity, and subjective masculinity stress: A moderated mediation model. *Journal of Counseling Psychology*, 61(4), 560–569. DOI: 10.1037/cou0000038 |
|  |  |  |  |  |  | Hopkins Symptom Checklist-21-item version | Psychological distress |  |  |  |
| **92** | India (2017) | cross-sectional (observational) | university institute | M=23 | 177  (64.4%) | Likert scale stress questionnaire | stress indicators | males and boys (physiological and social) | Male and Female Engineering Students | Yadav, R., Khanna, A., & Singh, D. (2017). Exploration of Relationship Between Stress and Spirituality Characteristics of Male and Female Engineering Students: A Comprehensive Study. *Journal of Religion & Health*, 56(2), 388–399. DOI: 10.1007/s10943-015-0174-7 |
| **93** | Korea (2010) | case-control  (observational) | college | M=23.35 | 104  (100%) | life stress questionnaire | life stress level | males (physiological) | Korean male college students | You, J.-S., Park, J.-Y., & Chang, K.-J. (2010). A case-control study on the dietary taurine intake, nutrient status and life stress of functional constipation patients in Korean male college students. *Journal of Biomedical Science*, 17(1). DOI: 10.1186/1423-0127-17-S1-S41 |
| **94** | Japan (2020) | laboratory (experimental) | population | 20-24 | 6  (50%) | Self-reported stress (SRS) | Subjective stress | males (physiological) | young male drivers | Zhao, Y., Yamamoto, T., & Kanamori, R. (2020). Study of older male drivers’ driving stress compared with that of young male drivers. *Journal of Traffic & Transportation Engineering (English edition)*, 7(4), 467–481. DOI: 10.1016/j.jtte.2018.10.011 |
| **95** | China (2018) | cross-sectional (observational) | college | M=22.4 | 582  (100%) | Depression Anxiety Stress Scale-21 (DASS-21) | measure the negative emotional states of depression, anxiety, and stress | males (physiological) | Chinese male senior college students | Zou, P., Sun, L., Yang, W., Zeng, Y., Chen, Q., Yang, H., Zhou, N., Zhang, G., Liu, J., Li, Y., Ao, L., & Cao, J. (2018). Associations between negative life events and anxiety, depressive, and stress symptoms: A cross-sectional study among Chinese male senior college students. *Psychiatry Research*, 270, 26–33. DOI: 10.1016/j.psychres.2018.09.019 |

**Supplementary Document B: Full search strategy**

| **Web of Science** | (TI=(male* OR boy* OR men OR man OR masculin*) AND TI=(stress*) AND ALL=(adolescen* OR youth OR young person OR teen* OR child* OR young people OR young adult OR young OR CYP OR minor OR juvenile OR pupil* OR student* OR looked after) AND ALL=(epidemiolog* OR observation* OR experiment* OR prevalence OR measure* OR assessment OR survey OR intervention OR evaluation OR prevention OR trials OR RCT OR Randomized Control Trial OR Randomised Control Trial OR quasi-experimental OR case control OR cohort OR twin studies)) AND LANGUAGE: (English) |
| --- | --- |
| **CINAHL Plus** | "TI ( male* OR boy* OR men OR man OR masculin* ) AND TI stress* AND ( adolescen* OR youth OR young person OR teen* OR child* OR young people OR young adult OR young OR CYP OR minor OR juvenile OR pupil* OR student* OR looked after ) AND ( epidemiolog* OR observation* OR experiment* OR prevalence OR measure* OR assessment OR survey OR intervention OR evaluation OR prevention OR trials OR RCT OR Randomized Control Trial OR Randomised Control Trial OR quasi-experimental OR case control OR cohort OR twin studies ) Publication Year: 1991-2021; English Language AND Apply equivalent subjects on 2021-02-17 08:21 AM" |
| **Scopus** | ( TITLE ( male* OR boy* OR men OR man OR masculin* ) AND TITLE ( stress* ) AND ALL ( adolescen* OR youth OR young AND person OR teen* OR child* OR young AND people OR young AND adult OR young OR cyp OR minor OR juvenile OR pupil* OR student* OR looked AND after ) AND ALL ( epidemiolog* OR observation* OR experiment* OR prevalence OR measure* OR assessment OR survey OR intervention OR evaluation OR prevention OR trials OR rct OR randomized AND control AND trial OR randomised AND control AND trial OR quasi-experimental OR case AND control OR cohort OR twin AND studies ) ) AND ( LIMIT-TO ( LANGUAGE , "English" ) ) |
| **PubMED** | (((male*[Title] OR boy*[Title] OR men[Title] OR man[Title] OR masculin*[Title]) AND (stress*[Title])) AND (adolescen* OR youth OR young person OR teen* OR child* OR young people OR young adult OR young OR CYP OR minor OR juvenile OR pupil* OR student* OR looked after)) AND (epidemiolog* OR observation* OR experiment* OR prevalence OR measure* OR assessment OR survey OR intervention OR evaluation OR prevention OR trials OR RCT OR Randomized Control Trial OR Randomised Control Trial OR quasi-experimental OR case control OR cohort OR twin studies) |
| **Proquest** | ti(male* OR boy* OR men OR man OR masculin*) AND ti(stress*) AND (adolescen* OR youth OR young person OR teen* OR child* OR young people OR young adult OR young OR CYP OR minor OR juvenile OR pupil* OR student* OR looked after) AND (epidemiolog* OR observation* OR experiment* OR prevalence OR measure* OR assessment OR survey OR intervention OR evaluation OR prevention OR trials OR RCT OR Randomized Control Trial OR Randomised Control Trial OR quasi-experimental OR case control OR cohort OR twin studies) |
| **PsychINFO** | 1. (male* or boy* or men or man or masculin*).m_titl. 2. "stress*".m_titl. 3. (adolescen* or youth or young person or teen* or child* or young people or young adult or young or CYP or minor or juvenile or pupil* or student* or looked after).mp. [mp=title, abstract, heading word, table of contents, key concepts, original title, tests & measures, mesh] 4. (epidemiolog* or observation* or experiment* or prevalence or measure* or assessment or survey or intervention or evaluation or prevention or trials or RCT or Randomized Control Trial or Randomised Control Trial or quasi-experimental or case control or cohort or twin studies).mp. [mp=title, abstract, heading word, table of contents, key concepts, original title, tests & measures, mesh] 5. 1 and 2 and 3 and 4 6. limit 5 to (english language and yr="1991 -Current") |

**Supplementary Document C: Preferred Reporting Items for Systematic reviews and Meta-Analyses extension for Scoping Reviews (PRISMA-ScR) Checklist**

| **SECTION** | **ITEM** | **PRISMA-ScR CHECKLIST ITEM** | **REPORTED ON PAGE #** |
| --- | --- | --- | --- |
| **TITLE** | | | |
| Title | 1 | Identify the report as a scoping review. | 1 |
| **ABSTRACT** | | | |
| Structured summary | 2 | Provide a structured summary that includes (as applicable): background, objectives, eligibility criteria, sources of evidence, charting methods, results, and conclusions that relate to the review questions and objectives. | 1 |
| **INTRODUCTION** | | | |
| Rationale | 3 | Describe the rationale for the review in the context of what is already known. Explain why the review questions/objectives lend themselves to a scoping review approach. | 6 |
| Objectives | 4 | Provide an explicit statement of the questions and objectives being addressed with reference to their key elements (e.g., population or participants, concepts, and context) or other relevant key elements used to conceptualize the review questions and/or objectives. | 6 |
| **METHODS** | | | |
| Protocol and registration | 5 | Indicate whether a review protocol exists; state if and where it can be accessed (e.g., a Web address); and if available, provide registration information, including the registration number. | 7 |
| Eligibility criteria | 6 | Specify characteristics of the sources of evidence used as eligibility criteria (e.g., years considered, language, and publication status), and provide a rationale. | 7 |
| Information sources* | 7 | Describe all information sources in the search (e.g., databases with dates of coverage and contact with authors to identify additional sources), as well as the date the most recent search was executed. | 8 |
| Search | 8 | Present the full electronic search strategy for at least 1 database, including any limits used, such that it could be repeated. | 8 |
| Selection of sources of evidence† | 9 | State the process for selecting sources of evidence (i.e., screening and eligibility) included in the scoping review. | 9 |
| Data charting process‡ | 10 | Describe the methods of charting data from the included sources of evidence (e.g., calibrated forms or forms that have been tested by the team before their use, and whether data charting was done independently or in duplicate) and any processes for obtaining and confirming data from investigators. | 10 |
| Data items | 11 | List and define all variables for which data were sought and any assumptions and simplifications made. | 11 |
| Critical appraisal of individual sources of evidence§ | 12 | If done, provide a rationale for conducting a critical appraisal of included sources of evidence; describe the methods used and how this information was used in any data synthesis (if appropriate). | n/a |
| Synthesis of results | 13 | Describe the methods of handling and summarizing the data that were charted. | 11 |
| **RESULTS** | | | |
| Selection of sources of evidence | 14 | Give numbers of sources of evidence screened, assessed for eligibility, and included in the review, with reasons for exclusions at each stage, ideally using a flow diagram. | 10 |
| Characteristics of sources of evidence | 15 | For each source of evidence, present characteristics for which data were charted and provide the citations. | 41-50 |
| Critical appraisal within sources of evidence | 16 | If done, present data on critical appraisal of included sources of evidence (see item 12). | n/a |
| Results of individual sources of evidence | 17 | For each included source of evidence, present the relevant data that were charted that relate to the review questions and objectives. | 41-50 |
| Synthesis of results | 18 | Summarize and/or present the charting results as they relate to the review questions and objectives. | 12-21 |
| **DISCUSSION** | | | |
| Summary of evidence | 19 | Summarize the main results (including an overview of concepts, themes, and types of evidence available), link to the review questions and objectives, and consider the relevance to key groups. | 21-28 |
| Limitations | 20 | Discuss the limitations of the scoping review process. | 28 |
| Conclusions | 21 | Provide a general interpretation of the results with respect to the review questions and objectives, as well as potential implications and/or next steps. | 28 |
| **FUNDING** | | | |
| Funding | 22 | Describe sources of funding for the included sources of evidence, as well as sources of funding for the scoping review. Describe the role of the funders of the scoping review. | 29 |

JBI = Joanna Briggs Institute; PRISMA-ScR = Preferred Reporting Items for Systematic reviews and Meta-Analyses extension for Scoping Reviews.

* Where *sources of evidence* (see second footnote) are compiled from, such as bibliographic databases, social media platforms, and Web sites.

† A more inclusive/heterogeneous term used to account for the different types of evidence or data sources (e.g., quantitative and/or qualitative research, expert opinion, and policy documents) that may be eligible in a scoping review as opposed to only studies. This is not to be confused with *information sources* (see first footnote).

‡ The frameworks by Arksey and O’Malley (6) and Levac and colleagues (7) and the JBI guidance (4, 5) refer to the process of data extraction in a scoping review as data charting*.*

§ The process of systematically examining research evidence to assess its validity, results, and relevance before using it to inform a decision. This term is used for items 12 and 19 instead of "risk of bias" (which is more applicable to systematic reviews of interventions) to include and acknowledge the various sources of evidence that may be used in a scoping review (e.g., quantitative and/or qualitative research, expert opinion, and policy document).

*From:* Tricco AC, Lillie E, Zarin W, O'Brien KK, Colquhoun H, Levac D, et al. PRISMA Extension for Scoping Reviews (PRISMAScR): Checklist and Explanation. Ann Intern Med. 2018;169:467–473. [doi: 10.7326/M18-0850](http://annals.org/aim/fullarticle/2700389/prisma-extension-scoping-reviews-prisma-scr-checklist-explanation).
